# CXCR4 inhibition alleviates prostatic inflammation and pelvic pain via suppressing Th17 cell differentiation and oxidative stress in EAP mice

**DOI:** 10.7150/ijbs.124532

**Published:** 2026-01-22

**Authors:** Yongtao Hu, Yi Liu, Jialin Meng, Ruijie Hu, Wenming Ma, Wenlong Xu, Kun Tang, Xianchao Dou, Chaozhao Liang, Li Zhang, Jing Chen

**Affiliations:** 1 Department of Urology, the First Affiliated Hospital of Anhui Medical University, Hefei, Anhui, China.; 2 Institute of Urology, Anhui Medical University, Hefei, Anhui, China.; 3 Anhui Province Key Laboratory of Urological and Andrological Diseases Research and Medical Transformation, Anhui Medical University, Hefei, Anhui, China.

**Keywords:** chronic prostatitis/chronic pelvic pain syndrome, CXCR4, Th17 cell, oxidative stress, apoptosis

## Abstract

**Background:** Chronic prostatitis/chronic pelvic pain syndrome (CP/CPPS) represents a prevalent urological disorder characterized by urinary symptoms, persistent pelvic or perineal discomfort accompanied by intraprostatic leukocyte infiltration. The C-X-C chemokine receptor type 4 (CXCR4) is critically involved in mediating inflammatory responses. Nevertheless, the specific involvement of CXCR4 in the immunoinflammatory mechanisms underlying CP/CPPS pathogenesis remains poorly characterized.

**Methods:** The therapeutic efficacy of AMD3100, a CXCR4 antagonist, in CP/CPPS was evaluated in a murine model of experimental autoimmune prostatitis (EAP). The progression of EAP and T helper 17 (Th17) cell-mediated immune responses following AMD3100 intervention was assessed via HE staining, immunohistochemistry, immunofluorescence, quantitative polymerase chain reaction (qPCR), and flow cytometry. To unravel mechanistic insights into the role of CXCR4 in regulating Th17 cell differentiation, RNA sequencing, qPCR, and western blotting validation were conducted. In addition, histological staining, measurements of reactive oxygen species (ROS) and peroxidation markers, and co-culture assays were employed to assess the antioxidative effects of AMD3100 in prostate epithelial cells.

**Results:** AMD3100 significantly alleviated a series of symptoms of prostatitis in EAP mice. Meanwhile, inhibition of CXCR4 by AMD3100 could significantly decrease the proportion of Th17 cells and downregulate the elevated expression of both pro-inflammatory and Th17-associated cytokines in these mice. However, administration of IL-17A partially reversed the therapeutic effects of AMD3100, elevating oxidative stress biomarkers and promoting the apoptosis of prostate epithelial cells. Mechanistically, CXCR4 inhibition suppresses NF-κB activation, thereby inhibiting Th17 cell differentiation. Furthermore, integrated findings from both *in vitro* and *in vivo* studies demonstrated that aberrant NF-κB activation not only counteracted AMD3100-mediated suppression of Th17 cell differentiation but also exacerbated prostatic epithelial cell damage through amplified inflammatory responses, oxidative stress, and apoptosis.

**Conclusions:** CXCR4 presents a promising therapeutic target for CP/CPPS. Pharmacological blockade of CXCR4 with AMD3100 inhibits Th17 cell differentiation, consequently mitigating inflammatory infiltration and oxidative tissue injury in CP/CPPS.

## 1. Introduction

Chronic prostatitis/chronic pelvic pain syndrome (CP/CPPS), impacting approximately 15% of men globally, is a persistent urological disorder. Its clinical presentation involves a constellation of symptoms such as persistent pelvic discomfort, urinary voiding issues, psychological distress, and sexual impairment 1-3. Current therapeutic strategies, including antibiotics, α receptor blockers, and anti-inflammatory agents, remain limited in efficacy due to their inability to comprehensively target the disease's multifactorial pathophysiology, which encompasses neuroinflammatory, psychological, and immunological components 4, 5. Emerging evidence implicates that CP/CPPS may arise from immune dysregulation, oxidative tissue damage, and neuroendocrine abnormality, yet its precise etiology remains incompletely defined 6. Immune cells exert multifaceted functions not only within the tumor microenvironment but also act as central orchestrators in the initiation and progression of autoimmune disorders 7-10. Mechanistic investigations utilizing the experimental autoimmune prostatitis (EAP) model have established T helper 17 (Th17) cells as an important driver of inflammatory cascades in prostatic microenvironment dysregulation 11-14. Although various prostatitis models exist, the EAP model exhibits histopathological and behavioral characteristics more closely resembling human CP/CPPS 15. Furthermore, the nonobese diabetic (NOD) mouse strain is increasingly being adopted for EAP induction due to its higher modeling efficacy and more consistent disease phenotype compared to other murine strains 16. Comparative studies have demonstrated that NOD mice develop significantly more severe prostatic inflammation, tissue damage, and elevated levels of pro-inflammatory cytokines following immunization than C57BL/6 or BALB/c mice 17. Therefore, we employed the NOD-based EAP model to elucidate novel pathways and therapeutic targets in CP/CPPS. Th17 cell-derived cytokines, mainly including IL-17A and IL-22, perpetuate inflammatory cascades and nociceptive sensitization by recruiting neutrophils and activating macrophages, and may stimulate prostate afferent nerves 18. However, the upstream molecular triggers of Th17 cell differentiation in the prostate microenvironment, particularly chemokine-mediated crosstalk between prostatic epithelial cells and infiltrating leukocytes, remain incompletely characterized, significantly hindering the development of targeted immunomodulatory therapies.

As the signature cytokine of Th17 cells, IL-17A may act directly on prostate epithelial cells to perpetuate tissue damage. Emerging evidence indicates that IL-17A exacerbates oxidative stress (OS) by enhancing lipid peroxidation, elevating hydrogen peroxide levels, and promoting oxidative phosphorylation, while concurrently suppressing key antioxidant enzymes, including glutathione peroxidase (GPX) 19. In lung epithelial cells, IL-17A has been demonstrated to induce the generation of reactive oxygen species (ROS) and mitochondrial dysfunction, leading to lipid peroxidation and DNA impairment 20, 21. Equally important is the role of IL-17A in regulating apoptosis in epithelial cells. In gastric epithelium, IL-17A promotes caspase-dependent cell death, a phenomenon similarly observed in alveolar and vascular endothelial cells 22-24. Moreover, IL-17A has been implicated in exacerbating atherosclerotic plaque progression by sensitizing vascular smooth muscle cells to TNF-α/IFN-γ-induced apoptosis 25, 26. However, whether IL-17A drives OS and apoptosis in prostate epithelial cells remains largely unexplored in the context of CP/CPPS.

The C-X-C motif chemokine receptor 4 (CXCR4) governs key biological processes, including leukocyte trafficking, embryo development, and tissue remodeling 27, 28. Beyond its canonical role in these physiological processes, aberrant CXCR4 activation has been closely linked to inflammatory diseases, including neuroinflammation and glomerular diseases, where it disrupts redox equilibrium 29, 30. Recent studies have established CXCR4 as a key mediator of OS-driven renal pathology by promoting podocyte injury and fibrotic progression 31. Specifically, CXCR4 overexpression triggers OS in podocytes and activates the renin-angiotensin system, while its genetic ablation alleviates podocyte damage and suppresses mesangial cell activation 32. In the context of the prostate microenvironment, elevated CXCR4 expression correlates with inflammatory infiltrates in clinical prostate tissue, yet the mechanistic contributions of CXCR4 to pelvic pain and glandular dysfunction remain largely undefined 33. Intriguingly, preclinical studies indicate that CXCR4 not only modulates T cell homing and function but also activates intracellular pathways linked to OS, which may synergistically aggravate CP/CPPS progression 34-36. Despite the aforementioned findings, this hypothesis has not been rigorously tested in validated CP/CPPS models. AMD3100 (plerixafor), a bicyclam derivative and selective CXCR4 antagonist, has exerted promising therapeutic efficacy in several preclinical studies regarding autoimmune and inflammatory diseases 37-39. By competitively blocking the interactions between CXCR4 and its ligand CXCL12, AMD3100 inhibits chemotaxis of immune cells, disrupts inflammatory cytokine networks, and suppresses pathological angiogenesis. In allergic asthma, AMD3100 attenuated disease severity by reducing Th17 cell infiltration into the respiratory system and downregulating IL-17 production 40. Similarly, in acute silicosis models, CXCR4 blockade limited inflammatory neutrophil infiltration, suppressed extracellular trap generation, and attenuated fibrosis in the pulmonary parenchyma 41. However, the therapeutic potential of AMD3100 in modulating pelvic pain, restoring prostatic redox homeostasis, and preventing epithelial apoptosis has yet to be elucidated.

In the current study, we have employed an EAP mouse model that recapitulates key features of human CP/CPPS to examine whether AMD3100 attenuates pelvic pain and prostatic inflammation by concurrently suppressing Th17 cell differentiation, OS, and apoptosis. We further explored the interplay among these pathological processes by assessing the regulatory effect of CXCR4 on NF-κB signaling. Our findings elucidate the multifaceted roles of CXCR4 in CP/CPPS pathogenesis and identify AMD3100 as a therapeutic candidate capable of simultaneously mitigating immune dysregulation, oxidative damage, and pro-apoptotic signaling. These results propose a novel mechanistic framework and offer translational implications for the treatment of refractory chronic inflammatory disorders.

## 2. Materials and Methods

### 2.1 Animal experiments and reagents

All animal experiments were conducted under ethics approval (Protocol ID: IACUC-2502084) granted by the Animal Ethics Committee of the First Affiliated Hospital of Anhui Medical University. The EAP model serves as a key tool for elucidating the etiology and pathophysiological pathways in chronic prostatitis 42-44. Prostate-derived antigens (PAgs) were isolated through tissue homogenization of prostate glands from Sprague-Dawley rats. Following BCA-based protein determination, aliquoted PAgs were maintained at -80°C until experimental use. Male NOD mice were sourced from the Nanjing Biomedical Research Institute (Nanjing, China) and maintained under specific pathogen-free (SPF) conditions. For immunization protocol, PAgs were combined with complete Freundʼs adjuvant (CFA) at an equal volume ratio to form a stable emulsion. Subcutaneous administration was performed at two timepoints (day 0 and 14 post-immunization) with 300 μg antigen/adjuvant mixture distributed across four anatomical sites: interscapular region (50 µL), sacrococcygeal area (50 µL), and foot pad regions (25 µL each). The animal study was conducted in three independent experimental stages, with five mice per group in each stage. During the initial phase, EAP-induced mice were randomized to receive either AMD3100 therapy (5 mg/kg/day) or phosphate-buffered saline (PBS) vehicle control through daily intraperitoneal injections. This pharmacological intervention persisted throughout the 14-day experimental period. CD4^+^CCR6^+^ T lymphocytes were purified from splenic suspensions via ‌flow cytometry sorting, with acquired cell samples immediately processed for transcriptome sequencing. At the second stage, AMD3100-treated EAP mice were randomly assigned to two groups: the AMD3100-monotherapy group maintained baseline treatment as controls, while in the combination group, AMD3100 treatment was supplemented with daily intraperitoneal administration of recombinant IL-17A (60 μg/kg/day) for a week. In the final experimental phase, all groups underwent subcutaneous immunization for EAP model establishment. The therapeutic regimen involved three distinct groups: the monotherapy group received AMD3100 (5 mg/kg/day) for two weeks, whereas the combination group received AMD3100 (5 mg/kg/day) for two weeks with NF-κB activator 1 (5 mg/kg/day) added during the final week via intraperitoneal injection. The administration parameters, such as injection volume, timing schedule, and procedural protocols, were standardized across all groups to minimize variables. Detailed information on the main biochemical reagents and antibodies is listed in **Table S1, 2**.

### 2.2 Evaluation of chronic pelvic pain

Behavioral assessments focused on cutaneous hyperalgesia associated with referred visceral pain. Tactile allodynia was evaluated using von Frey filaments applied to the lower abdominal region adjacent to the prostate, a site corresponding to prostate innervation, following established protocols 43, 45. On day 28 after intervention, mice were first acclimated for 30 minutes in isolated transparent plastic chambers. Tactile stimuli were delivered using filaments with forces of 0.04, 0.16, 0.4, 1.0, and 4.0 g, with each filament applied 10 times per mouse (1-2 seconds per stimulation) and a 2-minute interval between applications to reduce sensory adaptation. Positive responses to stimulation included three distinct behaviors: (1) vigorous abdominal wall retraction, (2) rapid licking or scratching responses localized to the stimulated area, or (3) jumping response. Response frequency was quantified as the percentage of positive reactions relative to the total number of stimulations (e.g., 3 positive responses in 10 times = 30%).

### 2.3 Hematoxylin-eosin (HE) staining

Following 24 hours of fixation in a 4% paraformaldehyde solution, the collected prostate tissues were embedded in paraffin. For pathological evaluation of the tissues, HE staining was utilized. To ensure objectivity, inflammation scoring was performed in a blinded manner. All HE-stained sections from different experimental groups were assigned random codes by an independent researcher not involved in the evaluation process. Two independent pathologists who were unaware of the group assignments then examined the sections under a light microscope. The criteria for evaluating prostatic inflammation have been described previously 17. Histopathological changes were scored on a scale of zero to three: zero indicated no obvious inflammation; one or two represented mild or moderate perivascular cuffing with mononuclear cell infiltration, respectively; and three represented severe perivascular cuffing, hemorrhage, and extensive mononuclear cell infiltration within the parenchyma. Representative photomicrographs for each score are provided in **Fig. S1** for visual reference. Five sections per group (one from each animal) were assessed. For each section, a representative microscopic field reflecting the overall inflammatory status was selected for scoring. A consensus score was reached by the two pathologists for each section and recorded as the final inflammation score for the corresponding animal.

### 2.4 Immunohistochemistry

Deparaffinized paraffin-embedded prostate tissue sections underwent antigen retrieval. Subsequently, endogenous peroxidase activity was blocked through incubating sections in 3% H_2_O_2_ at room temperature (RT) in the dark for 15 min. Then, after incubation with 3% BSA, the sections were probed with the primary antibody at a suitable dilution overnight at 4°C. Later on, the sections were extensively washed three times and subjected to the corresponding HRP-conjugated secondary antibody for 60 min at RT. Following extensive washing, the sections were dried and subjected to DAB chromogenic solution treatment. The reaction was terminated by rinsing the sections under running water. Hematoxylin was used for counterstaining to visualize the cell nuclei. Eventually, the sections were dehydrated and sealed, followed by observation under a microscope.

### 2.5 Immunofluorescence

Paraffin-embedded tissue slides were dewaxed and underwent antigen retrieval using citrate buffer. Subsequent to antigen blocking, the sections were probed with the primary antibody diluted at a suitable concentration overnight at 4°C. Then, the slides were treated with the corresponding secondary antibody in the dark for 2h at RT. DAPI was employed to stain the cell nuclei for 10 min incubation in the dark at RT. Finally, the sections were visualized and imaged via fluorescence microscopy.

### 2.6 Quantitative polymerase chain reaction (qPCR)

Total RNA was extracted from cultured cells and prostate tissues utilizing the TRIzol reagent. Following cDNA synthesis with All-in-One RT EasyMix, a commercially available reverse-transcription kit, qPCR was performed using SYBR Green Master Mix. Primer sequences are detailed in **Table S3**.

### 2.7 ROS detection analysis

Prostatic ROS levels were assessed via dihydroethidium (DHE) staining of fresh-frozen sections. Incubation proceeded at RT for 30 min within a humidified, light-protected chamber following the manufacturer's protocol. Fluorescence microscopy was employed for image acquisition. Intracellular ROS levels in prostate epithelial cells were analyzed using a ROS assay kit according to the manufacturer's instructions. Cells were incubated with diluted DCFH-DA, an oxidation-sensitive fluorescent probe, at RT for 20 min in 5% CO₂. After washing with serum-free medium, fluorescence was visualized immediately using a fluorescence microscope.

### 2.8 Assessment of malondialdehyde (MDA)

Prostate tissue samples and cells were homogenized. Then, the lysates were subjected to centrifugation at 12,000g for 10 min at 4 °C to obtain the supernatant. The protein concentration was evaluated via the bicinchoninic acid (BCA) assay, followed by the detection of MDA levels using the commercial Lipid Peroxidation MDA Assay Kit.

### 2.9 Western blotting

RIPA lysis solution was used to extract protein. Prior to electrophoresis, protein quantification was performed using the BCA assay. The quantified protein samples were subsequently combined with loading buffer and denatured at 95 °C for 10 min. The samples were then subjected to SDS-PAGE gel, and the proteins were transferred to a polyvinylidene difluoride (PVDF) membrane. The membrane was blocked using 5% nonfat milk and allowed to react with the primary antibody overnight at 4 °C. After extensive washing, the membranes were incubated with the secondary antibody for 1 h at RT. Following another extensive washing, the protein bands were finally visualized and imaged on the ChemiScope 5600 chemiluminescence system using the ECL luminescent reagent. The optical densities (OD) were analyzed with Image J software.

### 2.10 Flow cytometry analysis

The proportion of Th17 cells was quantified through flow cytometric analysis. Mouse splenic lymphocytes were harvested and subjected to two rounds of washing with PBS. The prepared cellular suspension was aliquoted into experimental tubes and exposed to FITC-conjugated CD4 surface markers for membrane antigen labeling. After subsequent PBS washing, cellular activation was induced through 4-hour incubation at 37°C with 1640 culture medium (Gibco, USA) supplemented with PMA, ionomycin, and monensin. Post-stimulation processing involved sequential cellular fixation and membrane permeabilization procedures. Intracellular staining was performed using PE-conjugated IL-17A antibodies through low-temperature incubation at 4 °C for 60 min. After the final PBS washing cycles, cellular fluorescence signals were captured using a Beckman Coulter flow cytometer, with subsequent data interpretation conducted through the CytExpert analysis platform.

### 2.11 Naïve CD4^+^ T cell isolation and differentiation

Naïve CD4^+^ T lymphocytes were isolated from murine splenocytes via magnetic-activated cell sorting technology using the Naïve CD4^+^ T cell Isolation Kit. Single-cell suspensions were sequentially incubated with Biotin-Antibody Cocktail, Anti-Biotin MicroBeads, and CD44 MicroBeads. Unlabeled cells were isolated via LS Column (Miltenyi Biotec, Germany), and the purity (> 95%) of harvested cells was verified before use. Purified naïve T cells were seeded in 24-well plates containing RPMI-1640 complete medium supplemented with 10% fetal bovine serum and 1% penicillin/streptomycin under standard conditions for a 5-day differentiation period. Th17 cell differentiation was induced using cytokine cocktails comprising 10 μg/mL anti-CD3 and 10 μg/mL anti-CD28 antibodies for T-cell activation, anti-IL-4 and anti-IFN-γ neutralizing antibodies (20 μg/mL of each antibody) for cytokine blockade, and polarizing factors including 1 ng/mL TGF-β1 combined with 40 ng/mL IL-6 and IL-23. Pharmacological interventions consisted of either 5 μg/mL AMD3100 added at culture initiation on day 0 or 1 μM NF-κB activator 1 administered during the late differentiation phase on day 4 46, 47, and all groups were cultured under identical conditions for 5 days.

### 2.12 Isolation of Th17 cells and RNA sequencing

Th17 cell populations were isolated via fluorescence-activated cell sorting using a FACSCalibur flow cytometer (BD Biosciences, Franklin Lakes, USA) through dual-labeling with FITC-conjugated anti-CD4 and PE-conjugated anti-CCR6 antibodies. Splenic lymphocytes underwent surface marker staining at RT for 60 min prior to sorting, with post-sort purity verification consistently exceeding 95% viability. Total RNA was isolated from CD4^+^CCR6^+^ lymphocyte subsets following standard methods. Illumina^®^ sequencing libraries were constructed with the NEBNext^®^ Ultra™ RNA Library Prep Kit. Complementary DNA synthesis utilized dNTP as substrates for second-strand synthesis catalyzed by DNA polymerase I. AMPure XP beads facilitated PCR product purification. The Illumina NovaSeq 6000 platform was employed to conduct the high-throughput RNA sequencing. Bioinformatics analyses implemented the DESeq2 algorithm for transcript quantification, with differential expression thresholds set at *P* < 0.05 and absolute log₂ (fold change) > 1.

### 2.13 Cell co-culture

Mouse prostate epithelial cells were purchased from Procell (CP-M064, Wuhan, China). A co-culture experiment was conducted using a transwell chamber system (Corning, 3401, USA). Th17 cells were generated using standard induction protocols with or without supplementation with 5 μg/mL AMD3100 and 1 μM NF-κB activator 1. Then, Th17 cells were washed three times with PBS and plated in the upper compartments, and mouse prostate epithelial cells were cultured in the lower chambers. This configuration maintained the cell populations in separate compartments while allowing the exchange of soluble factors. Following 24-hour incubation, prostate epithelial cells were harvested for functional analysis and protein extraction.

### 2.14 Statistical analysis

Each experimental group included five mice. Western blotting and cell culture assays were conducted with three independent replicates. Data were reported as the mean ± standard deviation (mean ± SD). A two-tailed Student's *t*-test was used for comparisons between two groups, whereas one- or two-way ANOVA with Tukey's post hoc test was employed for multi-group comparisons. Parametric tests were applied for data with normal distribution and homogeneous variance; otherwise, non-parametric tests were used. For RNA-seq analysis, differentially expressed genes (DEGs) between the EAP and EAP + AMD3100 groups were defined as those with an absolute log₂(fold change) > 1 and a *P* value < 0.05. Statistical analyses and graph generation were carried out using GraphPad Prism 9.0, R-4.0.2, SPSS 26.0, and Image J. The statistical significance was set at a *P* value < 0.05.

## 3. Results

### 3.1 AMD3100 alleviates prostatic inflammation and suppresses Th17 cell responses in EAP mice

**Figure 1:**
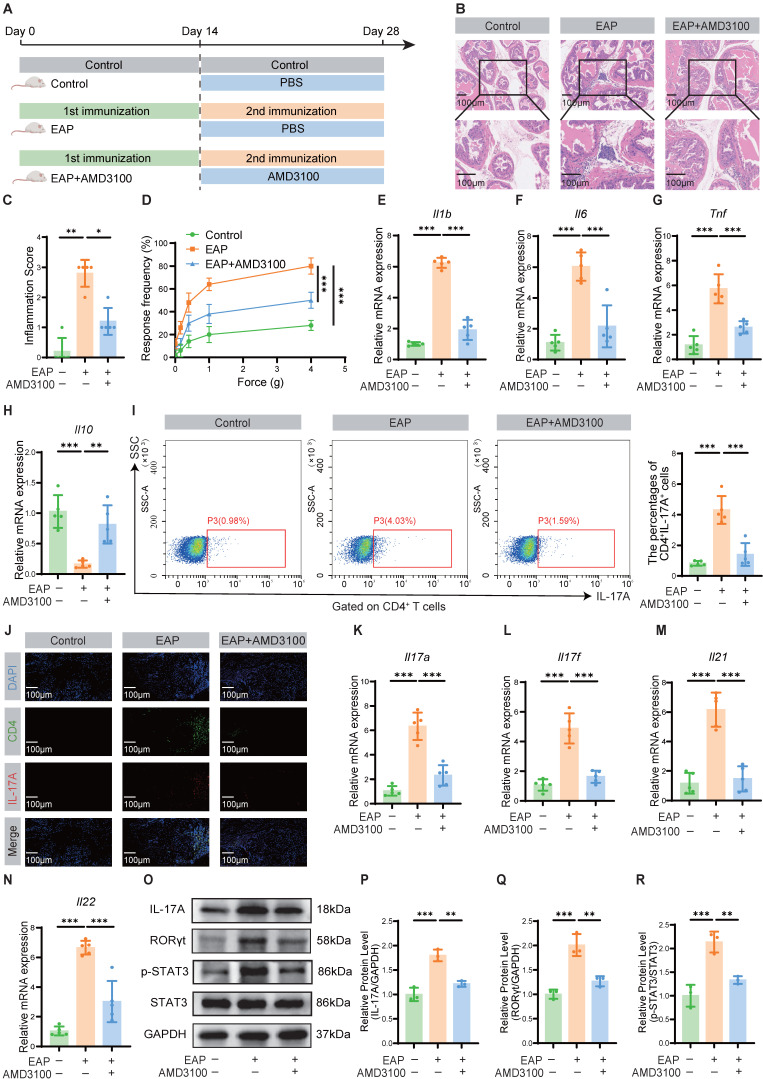
AMD3100 alleviates prostatic inflammation and suppresses Th17 cell responses in EAP mice. (A) A simple schematic diagram showed the whole process of AMD3100 treatment in EAP mice. (B) HE staining revealed the inflammatory cell infiltration within the prostate tissues in the control, EAP, and EAP + AMD3100 groups (n = 5). (C) Inflammation scores of the prostate tissues in the control, EAP, and EAP + AMD3100 groups (n = 5). (D) Differences in the pain responses of the mice in the control, EAP, and EAP + AMD3100 groups (n = 5). (E-H) qPCR analysis of the expression levels of inflammatory cytokines in the prostate tissues from the control, EAP, and EAP + AMD3100 groups (n = 5). (I) Proportion of CD4^+^IL-17A^+^ cells detected by flow cytometry in the splenic lymphocytes from the control, EAP, and EAP + AMD3100 groups (n = 5). (J) The infiltration of CD4^+^IL-17A^+^ cells in prostate tissues was determined by immunofluorescence in the control, EAP, and EAP + AMD3100 groups (n = 5). (K-N) qPCR analysis of the expression levels of Th17-associated cytokines in the prostate tissues from the control, EAP, and EAP + AMD3100 groups (n = 5). (O-R) The expression levels of IL-17A, RORγt, p-STAT3, and STAT3 in the prostate tissue from the control, EAP, and EAP + AMD3100 groups were detected by western blotting analysis (n = 3). *P < 0.05, **P < 0.01, ***P < 0.001. Th17: T helper 17; EAP: experimental autoimmune prostatitis; HE: hematoxylin-eosin; qPCR: quantitative polymerase chain reaction.

To assess the potential role of CXCR4 in EAP, we first examined its expression in prostate tissues. Our analysis revealed significant upregulation of CXCR4 at both the transcriptional and protein levels in EAP mice compared to controls (**Fig. S2**). Accordingly, we targeted this pathway using AMD3100, a specific CXCR4 antagonist, to determine its functional contribution to disease pathogenesis. A preliminary dose-response analysis identified 5 mg/kg as the optimal dose, achieving maximal therapeutic efficacy in alleviating inflammation and oxidative injury (**Fig. S3**). Therefore, this dose was selected for all subsequent mechanistic investigations. Following the secondary immunization, EAP mice were administered a two-week therapeutic regimen of AMD3100 (5 mg/kg) (**Fig. 1A**). Therapeutic outcomes were evaluated through quantitative assessment of histopathological inflammation, pelvic pain responses, and inflammatory cytokine profiles. Histopathological evaluation via HE staining demonstrated significant mitigation of inflammatory cell infiltration in prostate tissues of AMD3100-treated EAP mice compared with PBS-treated EAP mice (**Fig. 1B, C**). The therapeutic intervention markedly ameliorated mechanical hypersensitivity in EAP mice across a series of stimulus intensities (**Fig. 1D**). Quantitative analysis of inflammatory cytokine profiles revealed a marked elevation of *Il1b*, *Il6*, and *Tnf* transcripts in EAP mice relative to those in healthy mice, whereas AMD3100 administration substantially downregulated the corresponding mRNA levels of these pro-inflammatory genes (**Fig. 1E-G**). Notably, the mRNA expression of the anti-inflammatory gene *Il10* was downregulated in the EAP group, while pharmacological treatment with AMD3100 facilitated the restoration of *Il10* to baseline levels (**Fig. 1H**). To elucidate the specific cells targeted by AMD3100 in the treatment of EAP, we searched the Human Protein Atlas database (https://www.proteinatlas.org/) and found that CXCR4 was predominantly expressed in T cells within the prostate (**Fig. S4**). Given that Th17 cell-mediated immunity is known to play a critical role in EAP progression, we sought to investigate the immunomodulatory effects of AMD3100 on Th17 cells in EAP pathogenesis through comprehensive immune profiling. Flow cytometric evaluation demonstrated a marked reduction in the proportion of Th17 cells in AMD3100-treated EAP mice compared with PBS-treated EAP mice (**Fig. 1I**). Immunofluorescence staining revealed reduced Th17 cell infiltration in the prostate of the AMD3100 therapeutic group versus PBS-treated EAP mice (**Fig. 1J**). Transcriptomic profiling corroborated these findings, showing significant downregulation of Th17-associated genes (*Il17a*, *Il17f*, *Il21*, *Il22*) in AMD3100-treated mice (**Fig. 1K-N**). Western blotting further confirmed the diminished expression of IL-17A and key Th17 regulators, including RORγt and p-STAT3, in the prostatic lysates from the AMD3100 therapeutic group (**Fig. 1O-R**). These findings revealed that AMD3100 administration exerted dual therapeutic effects in the EAP mice, significantly suppressing prostatic inflammatory responses while concomitantly attenuating pelvic allodynia and modulating Th17-mediated immunopathology.

### 3.2 Exogenous IL-17A reverses the therapeutic effects of AMD3100 in EAP mice

**Figure 2:**
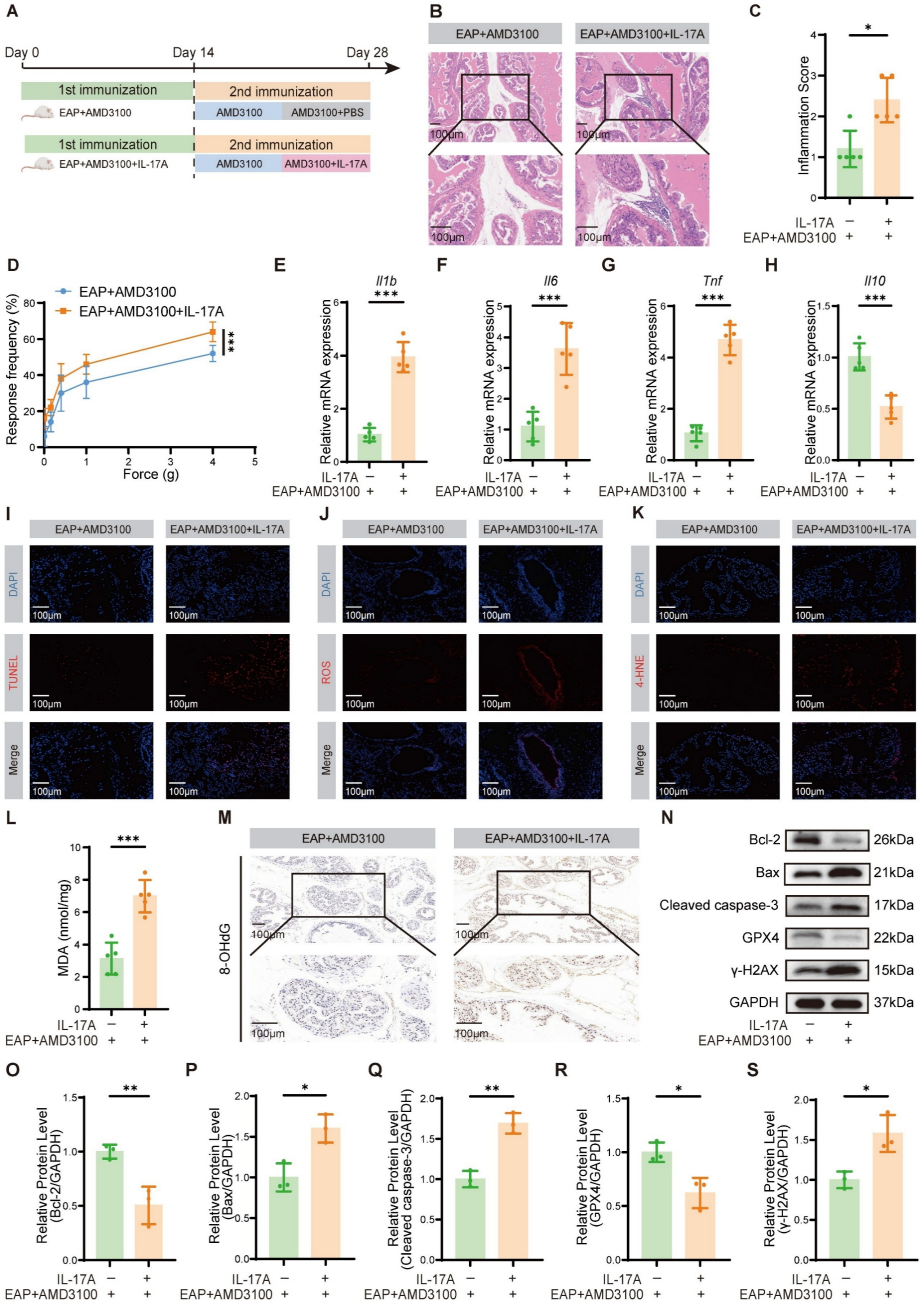
Exogenous IL-17A reverses the therapeutic effects of AMD3100 in EAP mice. (A) A simple schematic diagram showed the whole process of AMD3100 and IL-17A interventions in EAP mice. (B) HE staining revealed the inflammatory cell infiltration within the prostate tissues in the EAP + AMD3100 and EAP + AMD3100 + IL-17A groups (n = 5). (C) Inflammation scores of the prostate tissues in the EAP + AMD3100 and EAP + AMD3100 + IL-17A groups (n = 5). (D) Differences in the pain responses of the mice in the EAP + AMD3100 and EAP + AMD3100 + IL-17A groups (n = 5). (E-H) qPCR analysis of the expression levels of inflammatory cytokines in the prostate tissues from the EAP + AMD3100 and EAP + AMD3100 + IL-17A groups (n = 5). (I) TUNEL staining was used to assess the levels of apoptosis in the prostate tissues from the EAP + AMD3100 and EAP + AMD3100 + IL-17A groups (n = 5). (J) Immunofluorescence was used to assess the levels of ROS in the prostate tissues from the EAP + AMD3100 and EAP + AMD3100 + IL-17A groups (n = 5). (K) Immunofluorescence was used to assess the levels of 4-HNE in the prostate tissues from the EAP + AMD3100 and EAP + AMD3100 + IL-17A groups (n = 5). (L) The levels of MDA were measured in the prostate tissues from the EAP + AMD3100 and EAP + AMD3100 + IL-17A groups (n = 5). (M) Immunohistochemistry results for 8-OHdG in the prostate tissues from the EAP + AMD3100 and EAP + AMD3100 + IL-17A groups (n = 5). (N-S) The expression levels of Bcl-2, Bax, Cleaved caspase-3, GPX4, and γ-H2AX in the prostate tissues from EAP + AMD3100 and EAP + AMD3100 + IL-17A groups were detected by western blotting analysis (n = 3). *P < 0.05, **P < 0.01, ***P < 0.001. EAP: experimental autoimmune prostatitis; HE: hematoxylin-eosin; qPCR: quantitative polymerase chain reaction; ROS: reactive oxygen species; 4-HNE: 4-hydroxy-2-nonenal; MDA: malondialdehyde; 8-OHdG: 8-hydroxy-2'-deoxyguanosine; GPX4: glutathione peroxidase 4.

To determine whether the therapeutic efficacy of AMD3100 is mediated through suppression of Th17 cell function, we administered recombinant IL-17A to EAP mice following AMD3100 treatment. After a 7-day course of AMD3100, the mice received daily intraperitoneal injection of recombinant IL-17A for an additional 7 days (**Fig. 2A**). Histopathological analyses demonstrated that IL-17A administration counteracted the therapeutic benefits of AMD3100, by significantly reversing its suppressive effects on prostatic inflammatory cell infiltration (**Fig. 2B, C**) and pelvic allodynia (**Fig. 2D**). qPCR showed that exogenous IL-17A administration upregulated the mRNA expression of pro-inflammatory genes, including *Il1b*, *Il6* and *Tnf* (**Fig. 2E-G**), while suppressing the expression of anti-inflammatory gene *Il10* (**Fig. 2H**). Immunofluorescence staining further indicated an increase in apoptotic cell populations in prostatic tissues following IL-17A treatment (**Fig. 2I**). Given the established role of OS in CP/CPPS pathology, and the known pro-oxidative functions of IL-17A, we next explored whether IL-17A exacerbated oxidative injury in the prostate 48-50. MDA and 4-hydroxy-2-nonenal (4-HNE), which are commonly used lipid peroxidation markers, along with 8-hydroxy-2'-deoxyguanosine (8-OHdG), a marker of oxidative DNA damage, were assessed to quantify the redox imbalance. Immunofluorescence staining demonstrated that IL-17A significantly increased ROS production and 4-HNE accumulation (**Fig. 2J, K**), and the levels of MDA and 8-OHdG were also increased after IL-17A intervention (**Fig. 2L, M**). Western blotting analysis corroborated these findings, revealing increased expression of OS and apoptosis-related proteins in IL-17A-treated EAP mice (**Fig. 2N-S**). Collectively, these data indicated that exogenous IL-17A reversed the therapeutic effects of AMD3100 by reactivating pro-inflammatory and oxidative pathways. Mechanistically, IL-17A drove redox imbalance by promoting lipid peroxidation, oxidative DNA damage, and epithelial apoptosis, thereby highlighting its essential role in EAP progression.

### 3.3 AMD3100 inhibits Th17 cell differentiation *in vitro*

**Figure 3:**
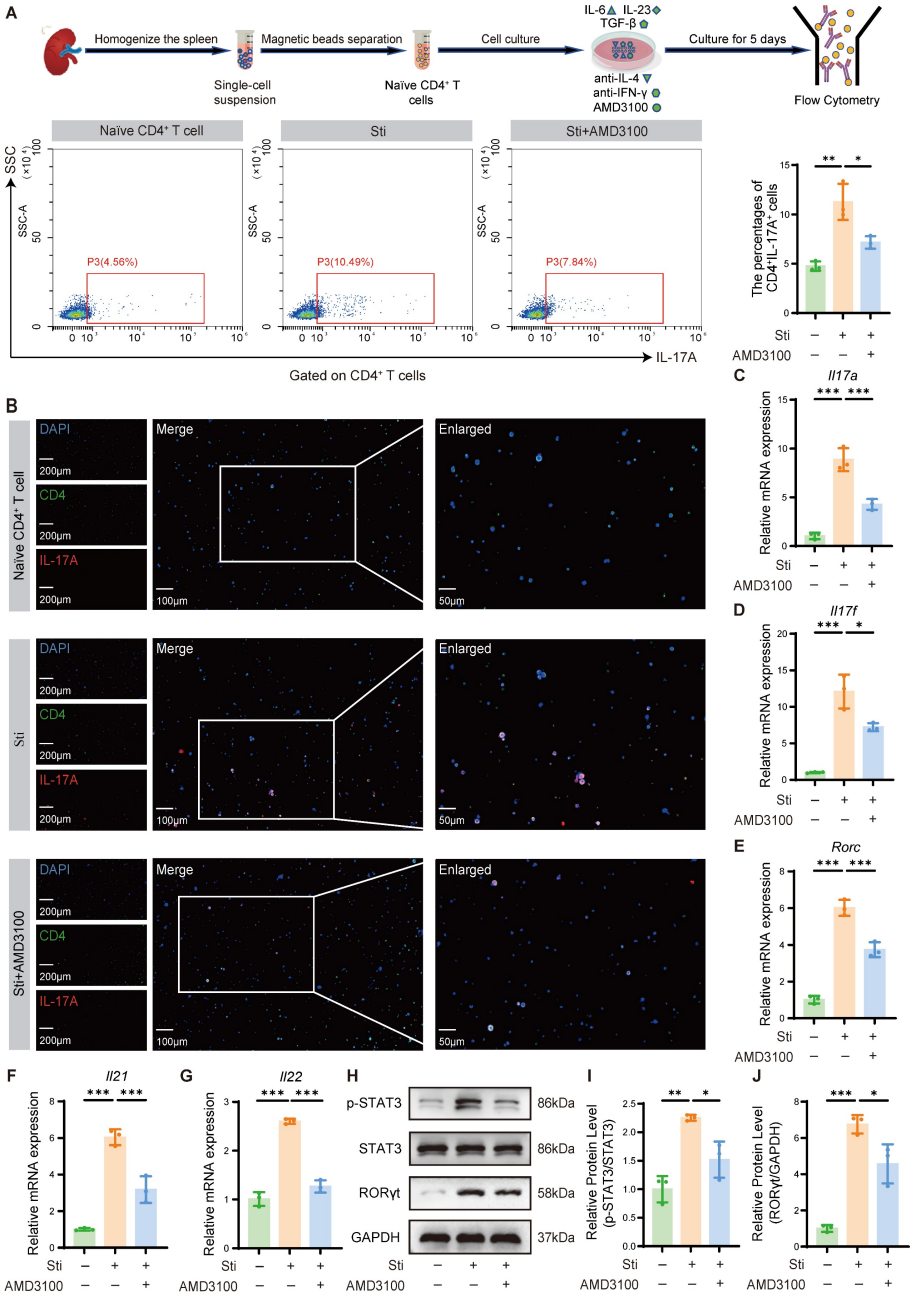
AMD3100 inhibits Th17 cell differentiation *in vitro*. (A) The *in vitro* Th17 cell differentiation ratio was determined by flow cytometry in the naïve CD4^+^ T cell, Sti, and Sti + AMD3100 groups (n=3). (B) The *in vitro* Th17 cell differentiation ratio was determined using immunofluorescence in the naïve CD4^+^ T cell, Sti, and Sti + AMD3100 groups (n = 3). (C-G) qPCR analysis of the expression levels of Th17-related genes in the naïve CD4^+^ T cell, Sti, and Sti + AMD3100 groups (n = 3). (H-J) The expression levels of p-STAT3, STAT3, and RORγt in the naïve CD4^+^ T cell, Sti, and Sti + AMD3100 groups were detected by western blotting analysis (n = 3). *P < 0.05, **P < 0.01, ***P < 0.001. Th17: T helper 17; Sti: stimulation with IL-6, IL-23, TGF-β1, anti-IFN-γ, and anti-IL-4; qPCR: quantitative polymerase chain reaction.

To mechanistically validate the immunomodulatory effects of AMD3100 observed *in vivo*, we established an *in vitro* Th17 polarization model to assess its impact on Th17 cell differentiation. Naïve CD4⁺ T cells were isolated by magnetic sorting and cultured under Th17-polarizing conditions using a cytokine cocktail consisting of IL-6, TGF-β1, and IL-23. Flow cytometric analysis revealed that AMD3100 impeded the differentiation of naïve CD4^+^ T cells into Th17 cells (**Fig. 3A**). These findings were further corroborated by immunofluorescence staining, which demonstrated decreased Th17 cell accumulation upon AMD3100 treatment (**Fig. 3B**). qPCR analysis showed that AMD3100 significantly downregulated the mRNA expression of Th17-related genes, including *Il17a*, *Il17f*, *Il21*, *Il22*, and *Rorc*, which encodes RORγt (**Fig. 3C-G**). Western blotting further confirmed that AMD3100 markedly inhibited the expression of the master transcription factor RORγt that transactivated IL-17A, and its upstream activator p-STAT3, both of which are critical regulators of Th17 cell differentiation (**Fig. 3H-J**). Cumulatively, these findings provide evidence that AMD3100 suppresses Th17 cell differentiation by downregulating transcriptional and signaling pathways essential for Th17 lineage development, thereby supporting its immunotherapeutic potential in Th17-driven inflammatory disorders.

### 3.4 The NF-κB signaling pathway is involved in the AMD3100-mediated inhibition of Th17 cell differentiation

**Figure 4:**
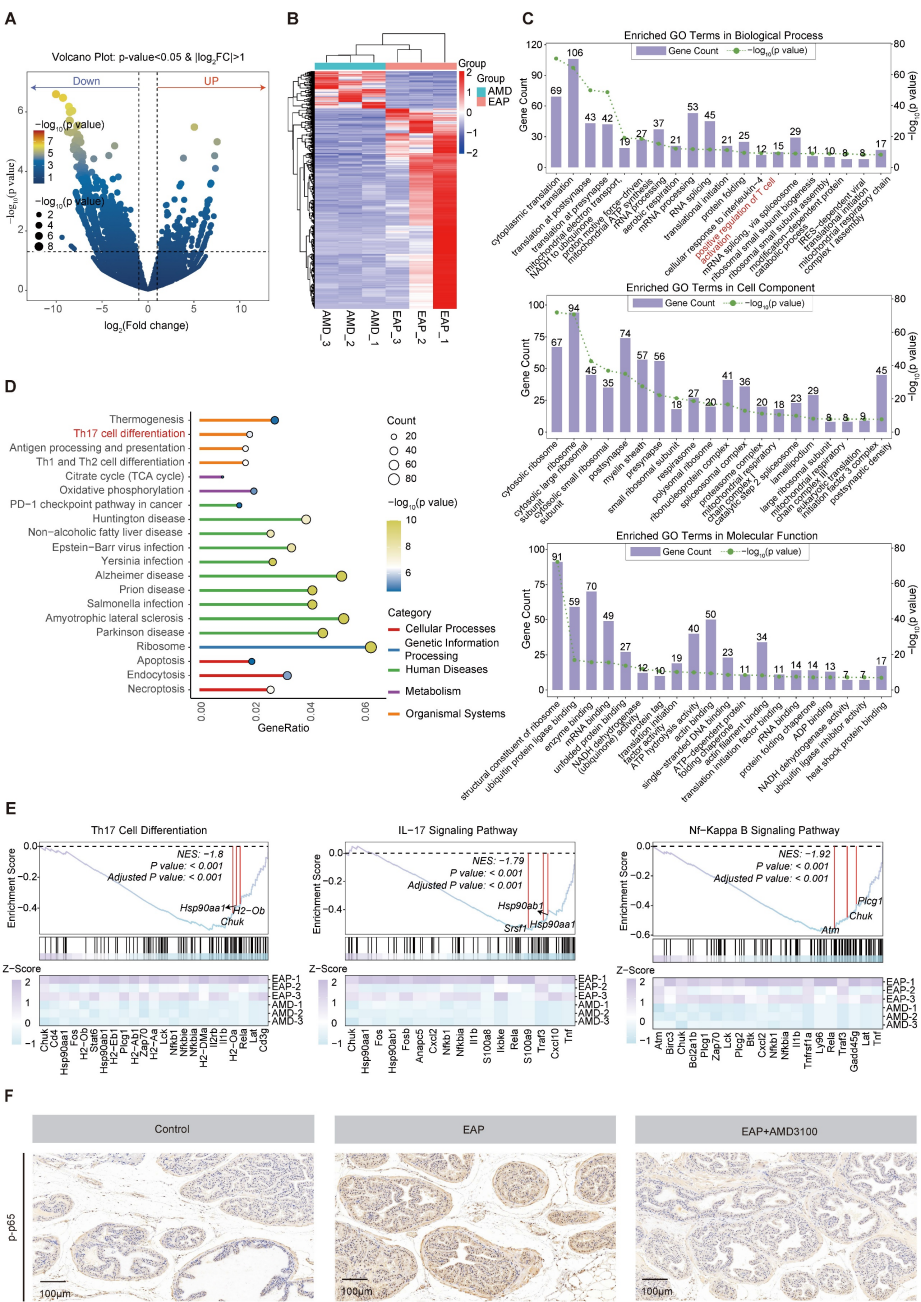
The NF-κB signaling pathway is involved in the AMD3100-mediated inhibition of Th17 cell differentiation. (A) Volcano plot showed differentially expressed genes in Th17 cells from mice in the EAP and EAP + AMD3100 groups. (B) The heatmap displayed the expression levels of differentially expressed genes in Th17 cells from mice in the EAP and EAP + AMD3100 groups. (C) GO enrichment analysis of differentially expressed genes. (D) KEGG pathway enrichment analysis of differentially expressed genes. (E) GSEA of Th17 cell differentiation, IL-17 signaling pathway, and NF-κB signaling pathway. (F) Immunohistochemistry results for p-p65 in the prostate tissues from the control, EAP, and EAP + AMD3100 groups (n = 5). Th17: T helper 17; EAP: experimental autoimmune prostatitis; GO: Gene Ontology; KEGG: Kyoto Encyclopedia of Genes and Genomes; GSEA: Gene Set Enrichment Analysis.

To reveal the underlying mechanisms by which AMD3100 modulates Th17 cell function, transcriptomic profiling of Th17 cells that were isolated from AMD3100-treated and PBS-treated EAP mice was performed. RNA sequencing analysis identified 1,576 DEGs consisted of 1,327 downregulated and 249 upregulated genes following AMD3100 intervention (**Fig. 4A**). The heatmap visualization of DEGs revealed distinct transcriptional signatures between groups (**Fig. 4B**). Subsequent Gene Ontology (GO) enrichment and Kyoto Encyclopedia of Genes and Genomes (KEGG) pathway enrichment analyses demonstrated significant enrichment of DEGs in pathways related to T cell activation and Th17 cell differentiation (**Fig. 4C, D**). Gene Set Enrichment Analysis (GSEA) further revealed a pronounced downregulation of gene sets associated with Th17 differentiation, IL-17 and NF-κB signaling pathway in AMD3100-treated mice compared to untreated EAP mice (**Fig. 4E**). Immunohistochemical staining corroborated these transcriptomic findings, showing elevated levels of phosphorylation level of p65 in the EAP model relative to control mice, which were markedly attenuated upon AMD3100 administration (**Fig. 4F**). Taken together, the therapeutic effects of AMD3100 in alleviating prostatitis, at least in part, are mediated through inhibition of NF-κB signaling, leading to impaired Th17 cell differentiation and downstream inflammatory responses.

### 3.5 NF-κB activation partially reverses the AMD3100-mediated inhibition of Th17 cell differentiation

**Figure 5:**
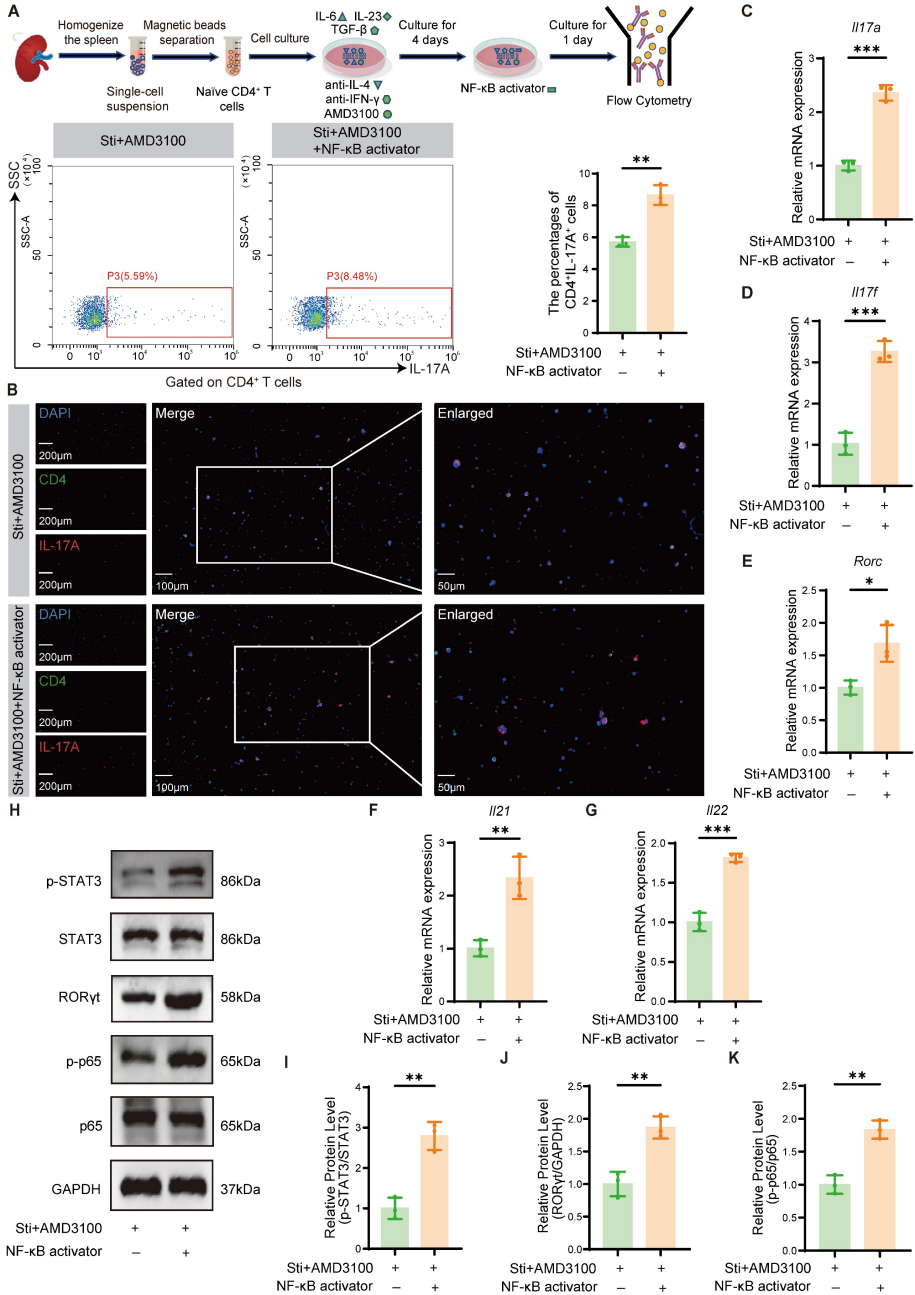
NF-κB activation partially reverses the AMD3100-mediated inhibition of Th17 cell differentiation. (A) The *in vitro* Th17 cell differentiation ratio was determined by flow cytometry in the Sti + AMD3100 and Sti + AMD3100 + NF-κB activator groups (n = 3). (B) The *in vitro* Th17 cell differentiation ratio was determined using immunofluorescence in the Sti + AMD3100 and Sti + AMD3100 + NF-κB activator groups (n = 3). (C-G) qPCR analysis of the expression levels of Th17-related genes in the Sti + AMD3100 and Sti + AMD3100 + NF-κB activator groups (n = 3). (H-K) The expression levels of p-STAT3, STAT3, RORγt, p-p65, and p65 in the Sti + AMD3100 and Sti + AMD3100 + NF-κB activator groups were detected by western blotting analysis (n = 3). *P < 0.05, **P < 0.01, ***P < 0.001. Th17: T helper 17; Sti: stimulation with IL-6, IL-23, TGF-β1, anti-IFN-γ, and anti-IL-4; qPCR: quantitative polymerase chain reaction.

To further delineate the mechanistic involvement of NF-κB signaling in AMD3100-mediated Th17 cell differentiation suppression, we investigated whether pharmacological activation of this pathway could counteract the inhibitory effects of AMD3100. Naïve CD4⁺ T cells were isolated and cultured under Th17-polarizing conditions with AMD3100, in the presence or absence of an NF-κB agonist. Flow cytometric analysis and immunofluorescence staining revealed that NF-κB activation significantly restored the differentiation of naïve CD4^+^ T cells into Th17 cells, reversing the suppressive effects of AMD3100 (**Fig. 5A, B**). qPCR analysis further demonstrated a substantial upregulation of Th17 lineage-specific transcripts, including *Il17a*, *Il17f*, *Rorc*, *Il21*, and *Il22*, in the Sti + AMD3100 + NF-κB activator group compared with the Sti + AMD3100 group (**Fig. 5C-G**). Western blotting analysis also showed elevated protein levels of RORγt and p-STAT3, two key transcriptional regulators of Th17 cell differentiation, upon NF-κB activation (**Fig. 5H-K**). In summary, these findings indicate that the suppressive effects of AMD3100 on Th17 cell development are partially mediated by downregulation of the NF-κB signaling pathway, highlighting its critical immunoregulatory impact on Th17-mediated inflammation.

### 3.6 AMD3100 attenuates OS damage and apoptosis in prostate epithelial cells

**Figure 6:**
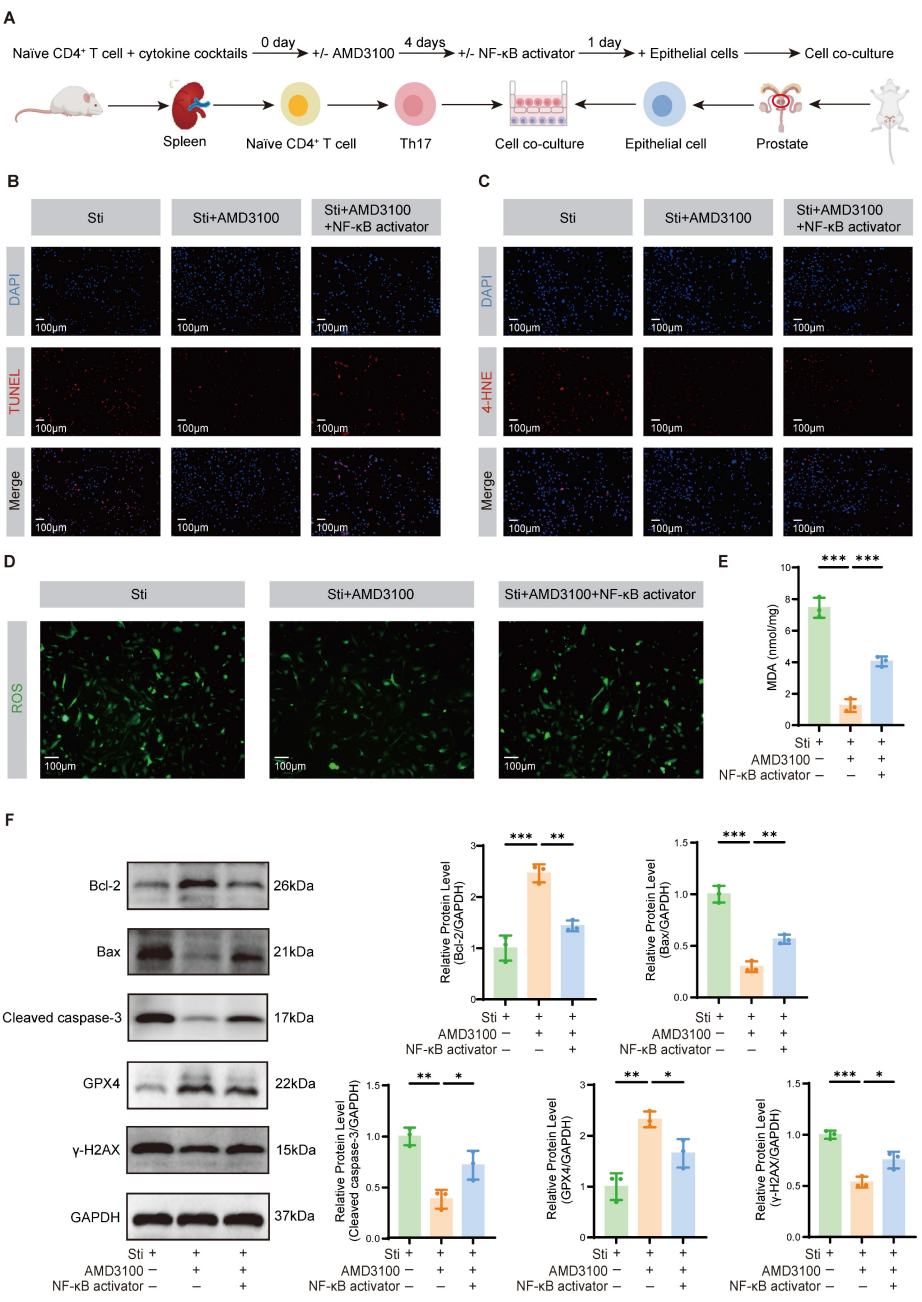
AMD3100 attenuates OS damage and apoptosis in prostate epithelial cells. (A) A simple schematic diagram of the co-culture system between Th17 cells and mouse prostate epithelial cells. (B) TUNEL staining was used to assess the levels of apoptosis in prostate epithelial cells from the Sti, Sti + AMD3100, and Sti + AMD3100 + NF-κB activator groups (n = 3). (C) Immunofluorescence was used to assess the levels of 4-HNE in prostate epithelial cells from the Sti, Sti + AMD3100, and Sti + AMD3100 + NF-κB activator groups (n = 3). (D) Immunofluorescence was used to assess the levels of ROS in prostate epithelial cells from the Sti, Sti + AMD3100, and Sti + AMD3100 + NF-κB activator groups (n = 3). (E) The levels of MDA were measured in prostate epithelial cells from the Sti, Sti + AMD3100, and Sti + AMD3100 + NF-κB activator groups (n = 3). (F) The expression levels of Bcl-2, Bax, Cleaved caspase-3, GPX4, and γ-H2AX in prostate epithelial cells from the Sti, Sti + AMD3100, and Sti + AMD3100 + NF-κB activator groups were detected by western blotting analysis (n = 3). *P < 0.05, **P < 0.01, ***P < 0.001. OS: oxidative stress; Th17: T helper 17; Sti: stimulation with IL-6, IL-23, TGF-β1, anti-IFN-γ, and anti-IL-4; 4-HNE: 4-hydroxy-2-nonenal; ROS: reactive oxygen species; MDA: malondialdehyde; GPX4: glutathione peroxidase 4.

To elucidate the impact of Th17 cells on OS-induced injury and apoptosis in prostate epithelial cells, we established a co-culture system as illustrated in **Fig. 6A**. Th17 cells were polarized *in vitro*, followed by their incubation with prostate epithelial cells to establish the inflammatory microenvironment. TUNEL staining revealed that the Sti + AMD3100 group had significantly less apoptosis of epithelial cells compared to the Sti group. In contrast, co-treatment with an NF-κB activator restored the pro-apoptotic capacity of Th17 cells, promoting epithelial cell death (**Fig. 6B**). Immunofluorescence staining demonstrated a marked reduction in 4-HNE accumulation in the Sti + AMD3100 group, whereas simultaneous NF-κB activation reversed this protective effect (**Fig. 6C**). A similar trend was observed in ROS staining, where intracellular ROS levels were notably decreased by AMD3100 but re-elevated upon NF-κB stimulation (**Fig. 6D**). Furthermore, measurement of MDA levels confirmed that AMD3100 significantly attenuated lipid peroxidation, while NF-κB activation markedly increased MDA concentrations (**Fig. 6E**). Western blotting analysis further substantiated these findings. The Sti + AMD3100 group had reduced the expression of Bax and Cleaved caspase-3, as well as the DNA damage marker γ-H2AX, while upregulating the Bcl-2 and antioxidant enzyme GPX4 (**Fig. 6F**). Collectively, these data indicate that AMD3100 mitigates Th17 cell-induced oxidative damage and epithelial apoptosis in the prostate by suppressing NF-κB signaling, underscoring its therapeutic potential in ameliorating redox imbalance and epithelial injury in CP/CPPS.

### 3.7 NF-κB activation partially reverses the therapeutic effects of AMD3100 in EAP mice

**Figure 7:**
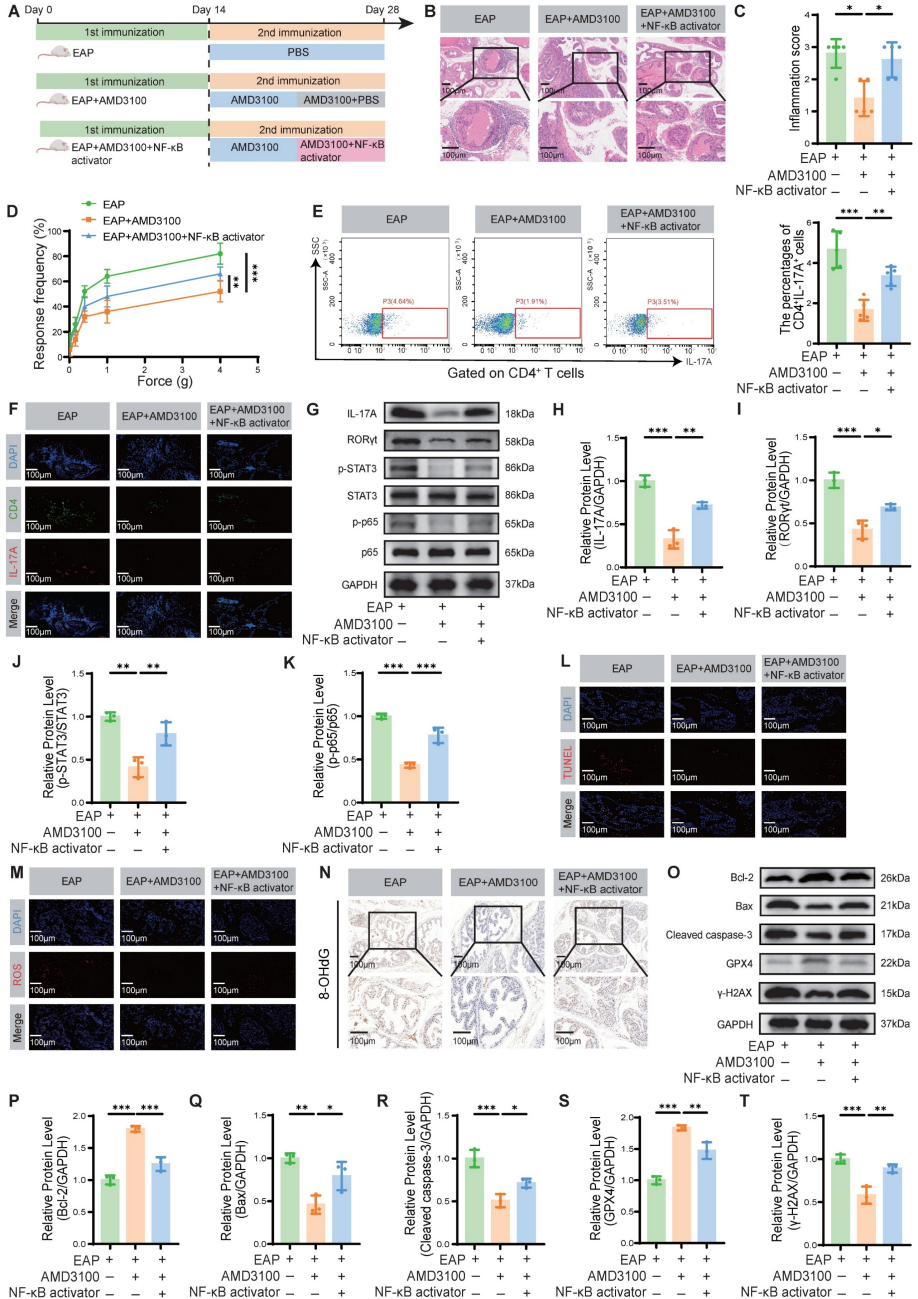
NF-κB activation partially reverses the therapeutic effects of AMD3100 in EAP mice. (A) A simple schematic diagram showed the whole process of AMD3100 and NF-κB activator interventions in EAP mice. (B) HE staining revealed the inflammatory cell infiltration within the prostate tissues in the EAP, EAP + AMD3100, and EAP + AMD3100 + NF-κB activator groups (n = 5). (C) Inflammation scores of the prostate tissues in the EAP, EAP + AMD3100, and EAP + AMD3100 + NF-κB activator groups (n = 5). (D) Differences in the pain responses of the mice in the EAP, EAP + AMD3100, and EAP + AMD3100 + NF-κB activator groups (n = 5). (E) Proportion of CD4^+^IL-17A^+^ cells detected by flow cytometry in the splenic lymphocytes from the EAP, EAP + AMD3100, and EAP + AMD3100 + NF-κB activator groups (n = 5). (F) The infiltration of CD4^+^IL-17A^+^ cells in prostate tissues was determined by immunofluorescence in the EAP, EAP + AMD3100, and EAP + AMD3100 + NF-κB activator groups (n = 5). (G-K) The expression levels of IL-17A, RORγt, p-STAT3, STAT3, p-p65, and p65 in the prostate tissue from the EAP, EAP + AMD3100, and EAP + AMD3100 + NF-κB activator groups were detected by western blotting analysis (n = 3). (L) TUNEL staining was used to assess the levels of apoptosis in the prostate tissues from the EAP, EAP + AMD3100, and EAP + AMD3100 + NF-κB activator groups (n = 5). (M) Immunofluorescence was used to assess the levels of ROS in the prostate tissues from the EAP, EAP + AMD3100, and EAP + AMD3100 + NF-κB activator groups (n = 5). (N) Immunohistochemistry results for 8-OHdG in the prostate tissues from the EAP, EAP + AMD3100, and EAP + AMD3100 + NF-κB activator groups (n = 5). (O-T) The expression levels of Bcl-2, Bax, Cleaved caspase-3, GPX4, and γ-H2AX in the prostate tissues from EAP, EAP + AMD3100, and EAP + AMD3100 + NF-κB activator groups were detected by western blotting analysis (n = 3). *P < 0.05, **P < 0.01, ***P < 0.001. EAP: experimental autoimmune prostatitis; HE: hematoxylin-eosin; ROS: reactive oxygen species; 8-OHdG: 8-hydroxy-2'-deoxyguanosine; GPX4: glutathione peroxidase 4.

**Figure 8:**
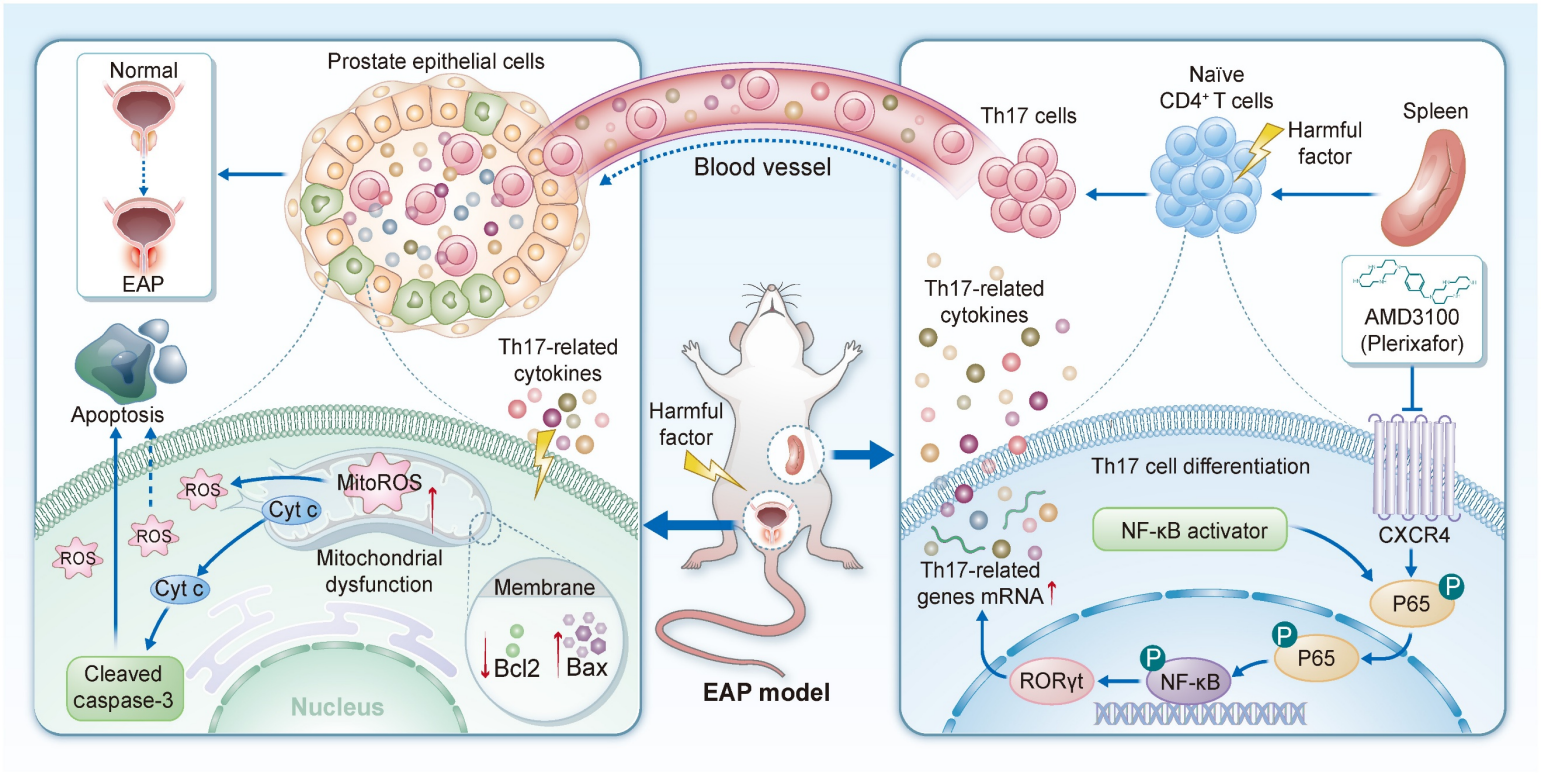
Schematic diagram illustrating the function and mechanism of AMD3100 in the treatment of EAP mice. EAP: experimental autoimmune prostatitis.

To determine whether AMD3100 exerts its therapeutic effects in EAP mice by inhibiting the NF-κB signaling pathway, thereby suppressing Th17 cell differentiation and subsequently attenuating OS and apoptosis in prostate epithelial cells, we administered an NF-κB activator to AMD3100-treated EAP mice (**Fig. 7A**). Histological analysis revealed that NF-κB activation significantly exacerbated prostatic inflammation, evidenced by increased inflammatory cell infiltration (**Fig. 7B, C**). Administration of the NF-κB activator significantly enhanced mechanical hypersensitivity in mice in response to stimuli of different forces (Fig. 7D). Compared with the EAP + AMD3100 group, the NF-κB activator-treated mice exhibited higher expression levels of proinflammatory genes *Il1b*, *Il6*, and *Tnf*, alongside a reduction in the anti-inflammatory gene *Il10* (**Fig. S5A-D**). Flow cytometry analysis of splenic lymphocytes revealed that NF-κB activator reversed the suppressive effect of AMD3100 on Th17 cell differentiation (**Fig. 7E**). Immunofluorescence staining further confirmed an increased infiltration of Th17 cells into prostate tissue upon simultaneous NF-κB activation (**Fig. 7F**). Consistent with these findings, qPCR analysis showed upregulated expression of Th17-related transcripts, including *Il17a*, *Il17f*, *Il21*, and *Il22*, in the prostate tissues of mice treated with the NF-κB activator (**Fig. S5E-H**). To clarify the protein expression profiles associated with Th17 cell differentiation, we conducted a western blotting analysis. The results indicated that the protein abundance of IL-17A, RORγt, p-STAT3, and p-p65 was significantly upregulated in the EAP + AMD3100 + NF-κB activator group (**Fig. 7G-K**). In parallel, TUNEL staining showed that the anti-apoptotic effect of AMD3100 was significantly reversed following NF-κB pathway activation (**Fig. 7L**). ROS and 4-HNE fluorescence staining showed that the NF-κB activator could effectively reverse OS in EAP mice prostate tissues (**Fig. 7M**; **Fig. S5I**), which was also confirmed in the expression of 8-OHdG (**Fig. 7N**) and MDA (**Fig. S5J**). Subsequent western blotting confirmed the consequences of NF-κB activation, with upregulation of Bax, Cleaved caspase-3, and γ-H2AX, and concomitant downregulation of Bcl-2 and GPX4 (**Fig. 7O-T**). Collectively, these results demonstrate that pharmacological activation of NF-κB signaling effectively negates the therapeutic benefits of AMD3100 in EAP mice by reactivating Th17 cell differentiation, enhancing OS, and promoting epithelial apoptosis (**Fig. 8**). These findings highlight the central role of the NF-κB/Th17 signaling axis in CP/CPPS pathogenesis and underscore the mechanistic basis of the immunomodulatory and cytoprotective effects executed by AMD3100.

## 4. Discussion

CP/CPPS is a clinically challenging and debilitating disorder characterized by chronic pelvic pain and lower urinary tract symptoms in the absence of identifiable infection. However, the underlying pathophysiological mechanisms remain incompletely elucidated. Growing evidence implicates dysregulated immune responses and OS as central contributors to disease development and progression. Nevertheless, the precise molecular interactions and regulatory networks linking these pathological processes have yet to be fully defined 6. We investigated the therapeutic efficacy of AMD3100 in the EAP model. Our results provide novel mechanistic insights and suggest the potential of CXCR4-targeted interventions in the treatment of CP/CPPS.

Administration of AMD3100 significantly attenuated prostatic inflammation and alleviated pelvic pain in EAP mice. This finding aligns with previous studies demonstrating the anti-inflammatory effects of AMD3100 in various models of inflammation and immune-mediated disorders 51-54. This therapeutic effect may be mediated by the blockade of a hyperactive CXCL12/CXCR4 axis. This premise is supported by our previous findings, which established a significant upregulation of CXCL12 in the prostates of EAP mice and in the serum of CP/CPPS patients 55. Therefore, the pathogenesis of CP/CPPS likely involves a cascade where upregulated CXCL12 binds to and activates CXCR4 on immune cells (such as Th17 cell precursors), promoting their differentiation, recruitment, and the subsequent inflammatory and oxidative damage. Our current study, which therapeutically targets CXCR4, functionally validates this pathway as a critical driver of disease. At the molecular level, treatment with AMD3100 resulted in a downregulated expression of pro-inflammatory genes, while concurrently restoring the expression of the anti-inflammatory gene. Our study demonstrates that pharmacological inhibition of CXCR4 effectively ameliorates prostatic inflammation and pain. Consistent with our findings, Huang et al. reported that AMD3100 effectively suppressed inflammatory cytokine expression in a murine model of focal cerebral ischemia 37. Similarly, Zgraggen et al. demonstrated that CXCL12/CXCR4 signaling plays a critical role in immune cell trafficking and pathological angiogenesis under inflammatory conditions, and that pharmacological blockade of this pathway attenuated cutaneous inflammation and reduced immune cell infiltration and neovascularization 56.

Comprehensive immunophenotyping revealed that AMD3100 treatment significantly suppressed Th17 cell responses. Th17 cells are well-established contributors to the pathogenesis of various inflammatory disorders, and their involvement in the progression of EAP has gained increasing recognition 11, 57, 58. Our findings provide evidence that AMD3100 modulates Th17-mediated immunopathology, thereby offering a novel therapeutic approach for prostatitis. These results align with those of Shang et al., who demonstrated that AMD3100 reduces Th17 cell proportion and related gene expression, thereby improving cardiac function in a mouse model of transverse aortic constriction under anti-cytotoxic T-lymphocyte-associated antigen 4 (CTLA-4) antibody therapy 59. Notably, the therapeutic effects of AMD3100 were reversed by exogenous administration of IL-17A, indicating that targeting Th17 cells represents a key mechanism underlying its efficacy. Further mechanistic analyses revealed that IL-17A administration not only abrogated the beneficial effects of AMD3100 but also exacerbated OS-related damage, as evidenced by increased lipid peroxidation (4-HNE, MDA), oxidative DNA damage (8-OHdG), and the expression of apoptotic markers. These pathological changes were associated with excessive ROS production and suppression of the antioxidant enzyme GPX4. Importantly, AMD3100 partially rescued the IL-17A-induced oxidative phenotype, suggesting that CXCR4 inhibition disrupts a pathogenic feed-forward loop in which Th17-derived IL-17A amplifies both inflammation and oxidative injury. This mechanism is particularly relevant in the context of CP/CPPS, where OS has been increasingly recognized as a key driver of therapeutic resistance and disease recurrence 60-62. Moreover, IL-17A has emerged as a crucial mediator linking chronic inflammation and oxidative damage 63, 64, further highlighting the potential of targeting the CXCL12/CXCR4/Th17 axis in the management of chronic prostatic inflammation.

*In vitro* experiments further elucidated the immunomodulatory effects of AMD3100 on Th17 cell differentiation. AMD3100 inhibited Th17 cell development, as evidenced by a reduced Th17 cell proportion and downregulated expression of related genes and proteins. These findings align with prior reports demonstrating that CXCR4 blockade by AMD3100 not only suppresses Th17 cell differentiation in both murine and human systems but also attenuates Th17 pathogenicity by reducing the secretion of pro-inflammatory mediators like IL-17, CCL3, CCL4, and TNF-α 46, 65. A recent study demonstrated that inhibiting the CXCR4 receptor could suppress STAT3 activation in hepatic CD4⁺ T cells, thereby blocking their differentiation into Th17 cells. Furthermore, treatment with AMD3100 could also inhibit STAT1 activation in these cells and impede Th1 cell differentiation 66. Despite these advances, the cellular and molecular mechanisms underlying CXCR4-mediated regulation of Th17 cells remain incompletely defined. Our study identifies the NF-κB signaling pathway as the downstream effector in this process. Transcriptomic (RNA-seq) and pathway enrichment analyses revealed that AMD3100 treatment significantly downregulated genes within key pathways driving Th17 cell pathogenicity, such as those involved in Th17 differentiation, IL-17 signaling, and NF-κB activation. The NF-κB pathway is a central regulator of immune responses and inflammation and has been implicated in the Th17 cell differentiation 67. Moreover, its constitutive activation is a key driver of progression in prostate diseases, making pharmacologic inhibition of this pathway a promising therapeutic strategy 68. Our data indicate that AMD3100 suppresses NF-κB activation and Th17 cell differentiation in EAP mice, contributing to the amelioration of prostatic inflammation and pelvic pain. Rescue experiments established the necessity of NF-κB suppression by showing that its pharmacological activation offset AMD3100-mediated inhibition of Th17 cell differentiation *in vitro*. Collectively, these findings identify the NF-κB signaling cascade as a critical downstream mediator of CXCR4 signaling in Th17 cell biology and a potential therapeutic target in chronic prostatitis. Beyond this immunomodulatory mechanism, emerging evidence indicates that the CXCL12/CXCR4 axis is a potent stimulus for pathological angiogenesis. Zgraggen et al. demonstrated that CXCR4 blockade with AMD3100 simultaneously curbed immune-cell infiltration and reduced neovascularisation in two murine models of chronic skin inflammation 56. Previous studies further support that CXCR4 directly promotes angiogenesis both *in vitro* and *in vivo*, and that pharmacological inhibition with AMD3100 can correct such aberrant vascular growth 69, 70. Given the clinical observations of microvascular proliferation and congestion in CP/CPPS, we speculate that the therapeutic benefits of AMD3100 may also involve the normalization of aberrant prostatic vasculature. Future studies aiming at quantifying vascular density, blood flow, and the expression of angiogenic factors in the EAP model following CXCR4 blockade will be crucial to test this hypothesis. Unraveling the potential contribution of CXCR4-driven angiogenesis to CP/CPPS pathogenesis could provide a more comprehensive understanding of the disease and further validate CXCR4 as a multifaceted therapeutic target.

Co-culture experiments further demonstrated that AMD3100 protected prostate epithelial cells from ROS-induced damage, with a significant reduction in apoptosis. Specifically, the presence of AMD3100 significantly reduced the pro-apoptotic and pro-oxidative effects mediated by Th17 cells, indicating that CXCR4 inhibition conferred the protective effects on prostate epithelial integrity. This observation is particularly relevant given the critical role of ROS and epithelial apoptosis in the pathogenesis of CP/CPPS. Notably, these protective effects were abrogated upon pharmacological NF-κB activation, further supporting the involvement of NF-κB in utilizing the function of AMD3100. *In vivo* NF-κB activation in EAP mice resulted in exacerbated prostatic inflammation, enhanced Th17 cell differentiation, increased OS, and elevated apoptotic signaling, collectively counteracting the therapeutic benefits conferred by AMD3100. These findings support a co-targeting strategy against CXCR4 and its key downstream effector NF-κB to enhance therapeutic efficacy in prostatitis. Despite these promising results, several limitations need to be raised. First, as our findings are based solely on the widely used EAP mouse model, they may not fully recapitulate the complexity, heterogeneity, and immunopathological nuances of human CP/CPPS. Future studies should validate these findings in alternative animal models on the path toward clinical translation. Second, although we identified NF-κB signaling as a critical function mediator of AMD3100, the precise molecular interactions and potential involvement of additional signaling pathways remain to be elucidated. Further mechanistic studies are necessary to further explore the downstream consequences of NF-κB inhibition in this context. Third, our investigation focused primarily on Th17 cells and their role in mediating prostatic inflammation. However, other immune cell populations may also contribute to the disease process and could be affected by CXCR4 blockade. A more comprehensive immunological assessment is warranted to explore the comprehensive impact of AMD3100 on the inflammatory microenvironment of the prostate.

## 5. Conclusion

In conclusion, this study elucidates the mechanism by which AMD3100 exerts its therapeutic effects in EAP mice. The findings suggest that AMD3100 exerts its benefits through suppression of Th17 cell differentiation and the NF-κB signaling pathway, thereby reducing prostatic inflammation, OS, and apoptosis. These findings not only advance the better understanding of CP/CPPS pathophysiology but also provide a rationale for repurposing CXCR4 antagonists as multifunctional therapeutics for chronic inflammatory disorders. Future studies may explore the clinical translation of these findings and investigate the potential of AMD3100 for the treatment of prostatitis.

## Supplementary Material

Supplementary figures and tables.

## Abbreviations

CP/CPPS: chronic prostatitis/chronic pelvic pain syndrome
CXCR4: C-X-C chemokine receptor type 4
EAP: experimental autoimmune prostatitis
Th17: T helper 17
qPCR: quantitative polymerase chain reaction
ROS: reactive oxygen species
OS: oxidative stress
GPX: glutathione peroxidase
PAgs: prostate-derived antigens
SPF: specific pathogen-free
CFA: complete Freundʼs adjuvant
PBS: phosphate-buffered saline
HE: hematoxylin-eosin
RT: room temperature
DHE: dihydroethidium
MDA: malondialdehyde
BCA: bicinchoninic acid
PVDF: polyvinylidene difluoride
OD: optical density
SD: standard deviation
4-HNE: 4-hydroxy-2-nonenal
8-OHdG: 8-hydroxy-2'-deoxyguanosine
DEGs: differentially expressed genes
GO: Gene Ontology
KEGG: Kyoto Encyclopedia of Genes and Genomes
GSEA: Gene Set Enrichment Analysis
CTLA-4: cytotoxic T-lymphocyte-associated antigen 4
