# Supplementary material for: CXCR4 inhibition alleviates prostatic inflammation and pelvic pain via suppressing Th17 cell differentiation and oxidative stress in EAP mice
Source: Int J Biol Sci. 2026 Jan 22;22(4):2027–46. doi: 10.7150/ijbs.124532 (PMC12905642; doi:10.7150/ijbs.124532)
Supplement: Supplementary file 1 — Supplementary figures and tables. [file ijbsv22p2027s1.pdf]

Table S1. Products used in this study.

Products	Catalog number	Manufacturer
Complete Freund's adjuvant	F5881	Sigma-Aldrich
AMD3100	110078-46-1	TargetMol
NF-κB activator 1	HY-134476	MedChemExpress
IL-17A	HY-P73174	MedChemExpress
IL-6	CG39	Novoprotein
TGF-β1	CA59	Novoprotein
IL-23	CS31	Novoprotein
RNA extraction kit	220011	Fastagen
SYBR master qPCR mix and qPCR kit	22204	Tolobio
Malondialdehyde activity assay kit	S0131S	Beyotime
TUNEL apoptosis assay kit	RCT-50R	Recordbio
ROS assay kit	S0033S	Beyotime
Dihydroethidium assay kit	S0063	Beyotime
BCA protein assay kit	GK10009	GlpBio
PMA	CS0001	MultiSciences
Ionomycin	CS0002	MultiSciences
Monensin	CS0004	MultiSciences
Naïve $CD4^+$ T cell isolation kit	130-104-453	Miltenyi Biotec
anti-CD3	BE0002	Bio X Cell
anti-CD28	BE0015-1	Bio X Cell
anti-IL-4	BE0045	Bio X Cell
anti-IFN-γ	BE0055	Bio X Cell
anti-CD4-FITC	553047	BD Biosciences
anti-IL-17A-PE	559502	BD Biosciences
anti-CCR6-PE	129804	Biolegend

Table S2. Antibodies used in WB, IHC and IF.

Name	Application	Catalog number	Manufacturer	Dilution ratio
anti-CXCR4	WB	CY5380	Abways	1:1000
anti-IL-17A	WB	DF6127	Affinity	1:1000
anti-RORγt	WB	ab207082	abcam	1:2000
anti-STAT3	WB	9139T	Cell Signaling Technology	1:1000
anti-phospho-STAT3	WB	9145T	Cell Signaling Technology	1:2000
anti-Bcl-2	WB	68103-1-Ig	Proteintech	1:1000
anti-Bax	WB	AF0120	Affinity	1:1000
anti-Cleaved caspase-3	WB	CY5501	Abways	1:1000
anti-GPX4	WB	DF6701	Affinity	1:1000
anti-γ-H2AX	WB	CY6572	Abways	1:1000
anti-p65	WB	8242T	Cell Signaling Technology	1:1000
anti-phospho-p65	WB	3033T	Cell Signaling Technology	1:1000
anti-GAPDH	WB	AF7021	Affinity	1:3000
anti-phospho-p65	IHC	CY6372	Abways	1:100
anti-8-OHdG	IHC	GB150115-50	Servicebio	1:200
anti-4-HNE	IF	bs-6313R	Bioss	1:200
anti-CD4	IF	GB15064-50	Servicebio	1:200
anti-IL-17A	IF	GB11110-1-50	Servicebio	1:200

Table S3. The primer sequences.

**Gene**	**Forward primer sequence (5 ' to 3 ')**	**Reverse primer sequence (5 ' to 3 ')**
Cxcr4	GACTGGCATAGTCGGCAATG	AGAAGGGGAGTGTGATGACAAA
Il1b	GCAACTGTTCCTGAACTCAACT	ATCTTTTGGGGTCCGTCAACT
Il6	TAGTCCTTCCTACCCCAATTTCC	TTGGTCCTTAGCCACTCCTTC
Tnf	GAGTGACAAGCCTGTAGCC	CTCCTGGTATGAGATAGCAAA
Il10	GCTCTTACTGACTGGCATGAG	CGCAGCTCTAGGAGCATGTG
Il17a	TCAGCGTGTCCAAACACTGAG	CGCCAAGGGAGTTAAAGACTT
Il17f	TGCTACTGTTGATGTTGGGAC	CAGAAATGCCCTGGTTTTGGT
Rorc	TCCACTACGGGGTTATCACCT	AGTAGGCCACATTACACTGCT
Il21	CGCCTCCTGATTAGACTTCG	GCCCCTTTACATCTTGTGGA
Il22	ATGAGTTTTTCCCTTATGGGGAC	GCTGGAAGTTGGACACCTCAA
Gapdh	AGGTCGGTGTGAACGGATTTG	GGGGTCGTTGATGGCAACA

Figure S1. Representative hematoxylin-eosin stained prostate tissue sections illustrating the 0-3 grading system for prostatic inflammation.

Figure S2. CXCR4 is upregulated in the prostate tissues of EAP mice. (A) qPCR analysis of the expression level of *Cxcr4* in the prostate tissues from the Control and EAP groups (n = 5). (B) The expression level of CXCR4 in the prostate tissues from the Control and EAP groups (n = 3). ***P* < 0.01. CXCR4: C-X-C chemokine receptor type 4; EAP: experimental autoimmune prostatitis; qPCR: quantitative polymerase chain reaction.

Figure S3. Dose-response analysis of AMD3100 treatment in EAP mice. (A) A simple schematic diagram showed the whole process of AMD3100 intervention at doses of 1, 5, and 10 mg/kg in EAP mice. (B) HE staining revealed the inflammatory cell infiltration within the prostate tissues in the Control, EAP, EAP + AMD3100 (1mg/kg), EAP + AMD3100 (5mg/kg), and EAP + AMD3100 (10 mg/kg) groups (n = 5). (C) Inflammation scores of the prostate tissues in the Control, EAP, EAP + AMD3100 (1mg/kg), EAP + AMD3100 (5mg/kg), and EAP + AMD3100 (10 mg/kg) groups (n = 5). (D) Differences in the pain responses of the mice in the Control, EAP, EAP + AMD3100 (1mg/kg), EAP + AMD3100 (5mg/kg), and EAP + AMD3100 (10 mg/kg) groups (n = 5). (E, F) Proportion of $CD4^+IL\text{-}17A^+$ cells detected by flow cytometry in the splenic lymphocytes from the Control, EAP, EAP + AMD3100 (1mg/kg), EAP + AMD3100 (5mg/kg), and EAP + AMD3100 (10 mg/kg) groups (n = 5). (G-J) qPCR analysis of the expression levels of inflammatory cytokines in the prostate tissues from the Control, EAP, EAP + AMD3100 (1mg/kg), EAP + AMD3100 (5mg/kg), and EAP + AMD3100 (10 mg/kg) groups (n = 5). (K) Immunofluorescence was used to assess the levels of ROS in the prostate tissues from the Control, EAP, EAP + AMD3100 (1mg/kg), EAP + AMD3100 (5mg/kg), and EAP + AMD3100 (10 mg/kg) groups (n = 5). (L) Immunofluorescence was used to assess the levels of 4-HNE in the prostate tissues from the Control, EAP, EAP + AMD3100 (1mg/kg), EAP + AMD3100 (5mg/kg), and EAP + AMD3100 (10 mg/kg) groups (n = 5). (M) The expression levels of GPX4 and γ-H2AX in the prostate tissues from Control, EAP, EAP + AMD3100 (1mg/kg), EAP + AMD3100 (5mg/kg), and EAP + AMD3100 (10 mg/kg) groups (n = 5). **P* < 0.05, ***P* < 0.01, ****P* < 0.001. EAP: experimental autoimmune prostatitis; HE: hematoxylin-eosin; qPCR: quantitative polymerase chain reaction; ROS: reactive oxygen species; 4-HNE: 4-hydroxy-2-nonenal; GPX4: glutathione peroxidase 4.

Figure S4. Analysis of single-cell expression of CXCR4 in the prostate using the Human Protein Atlas database. CXCR4: C-X-C chemokine receptor type 4.

Figure S5. NF-κB activator partially negates the beneficial effects of AMD3100 in EAP mice. (A-D) qPCR analysis of the expression levels of inflammatory cytokines in the prostate tissues from the EAP, EAP + AMD3100, and EAP + AMD3100 + NF-κB activator groups (n = 5). (E-H) qPCR analysis of the expression levels of Th17-associated cytokines in the prostate tissues from the EAP, EAP + AMD3100, and EAP + AMD3100 + NF-κB activator groups (n = 5). (I) Immunofluorescence was used to assess the levels of 4-HNE in the prostate tissues from the EAP, EAP + AMD3100, and EAP + AMD3100 + NF-κB activator groups (n = 5). (J) The levels of MDA were measured in the prostate tissues from the EAP, EAP + AMD3100, and EAP + AMD3100 + NF-κB activator groups (n = 5). $^{*}P < 0.05$, $^{**}P < 0.01$, $^{***}P < 0.001$. EAP: experimental autoimmune prostatitis; qPCR: quantitative polymerase chain reaction; Th17: T helper 17; 4-HNE: 4-hydroxy-2-nonenal; MDA: malondialdehyde.

Figure S1

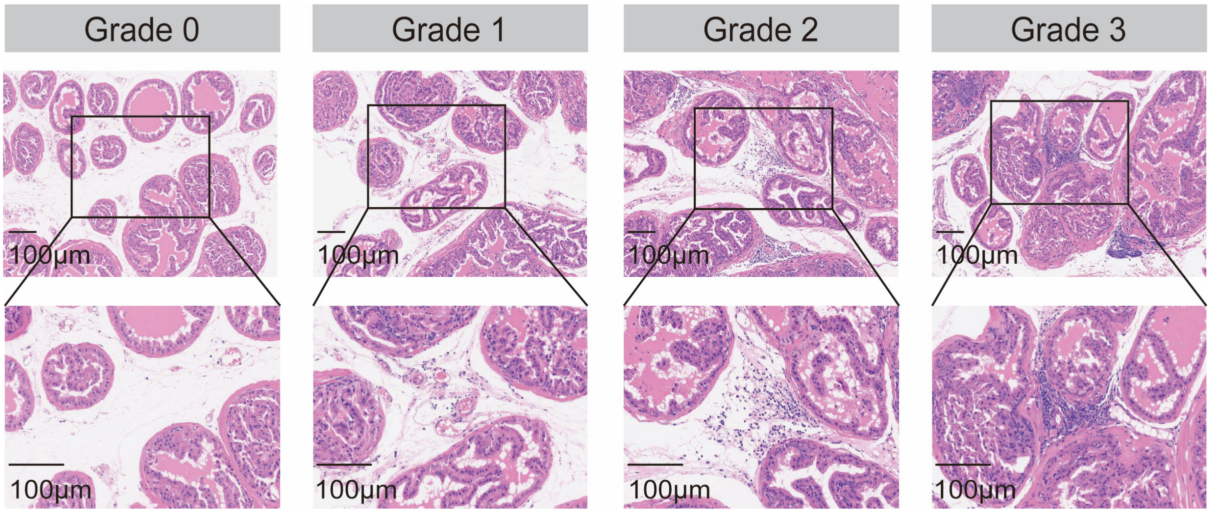

Figure S2

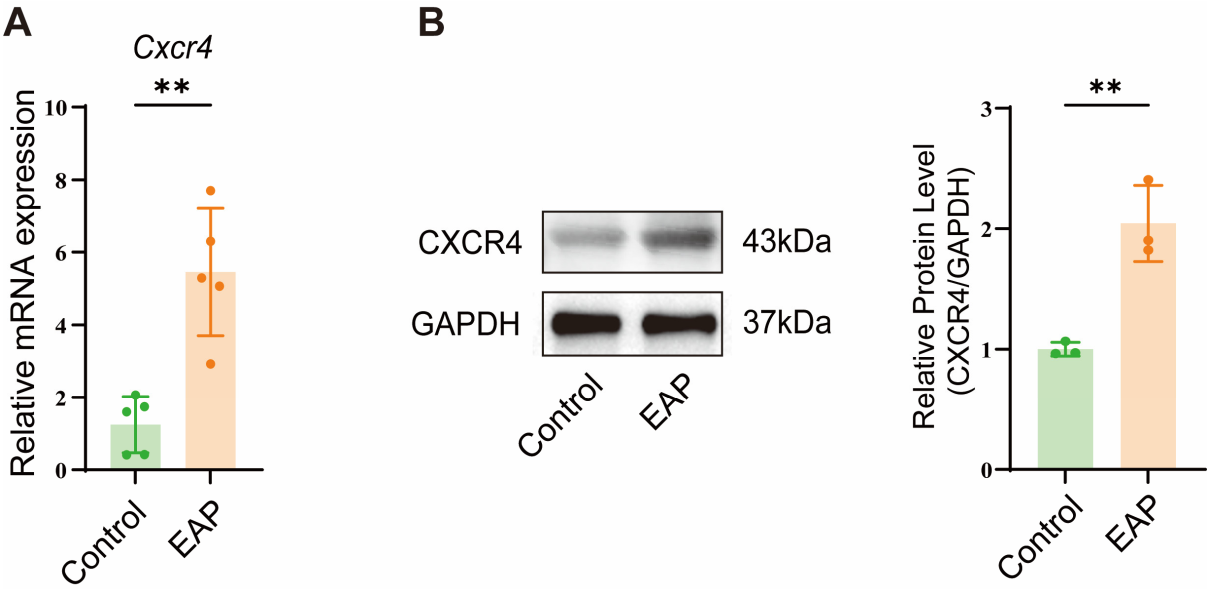

Figure S3

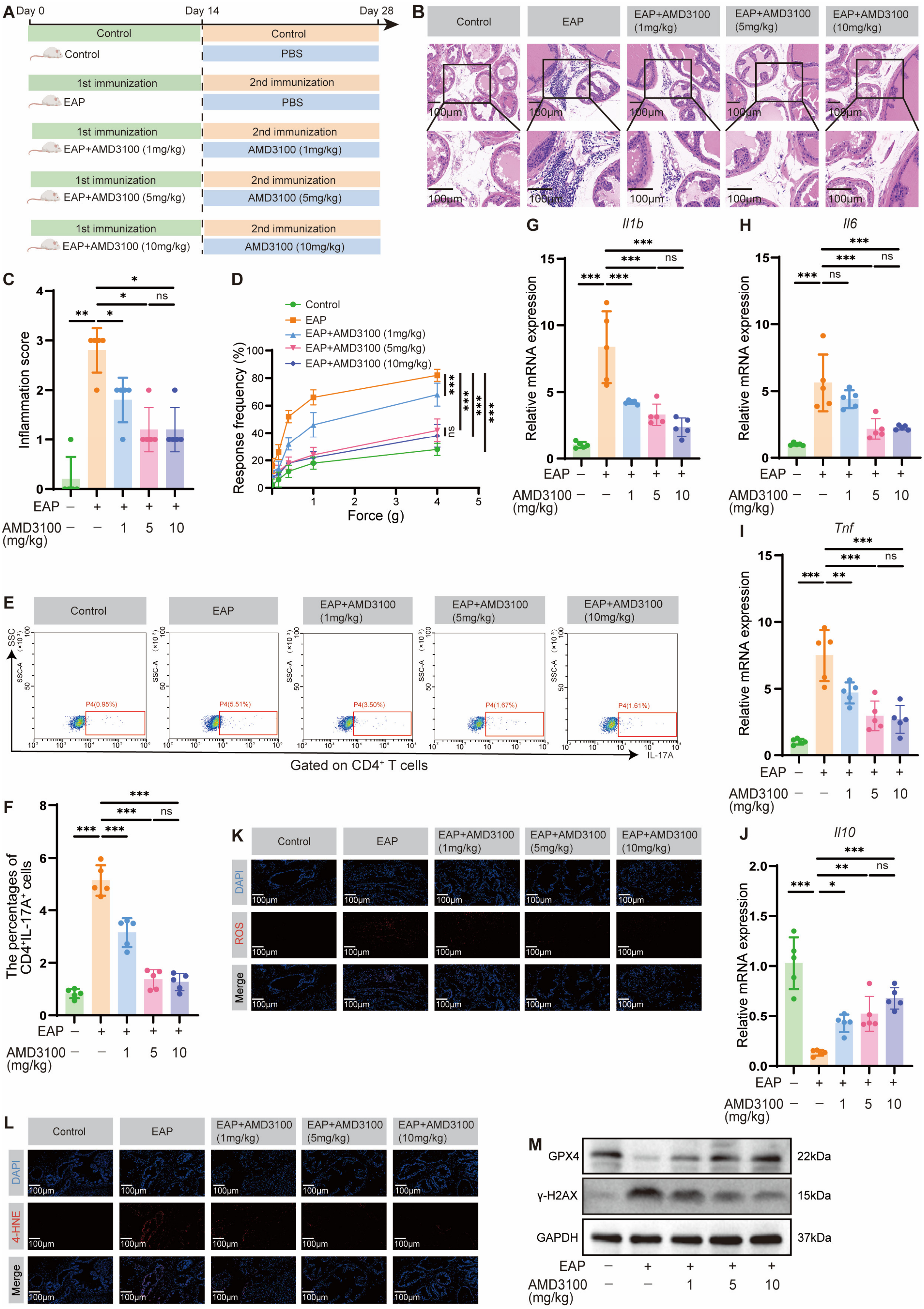

Figure S4

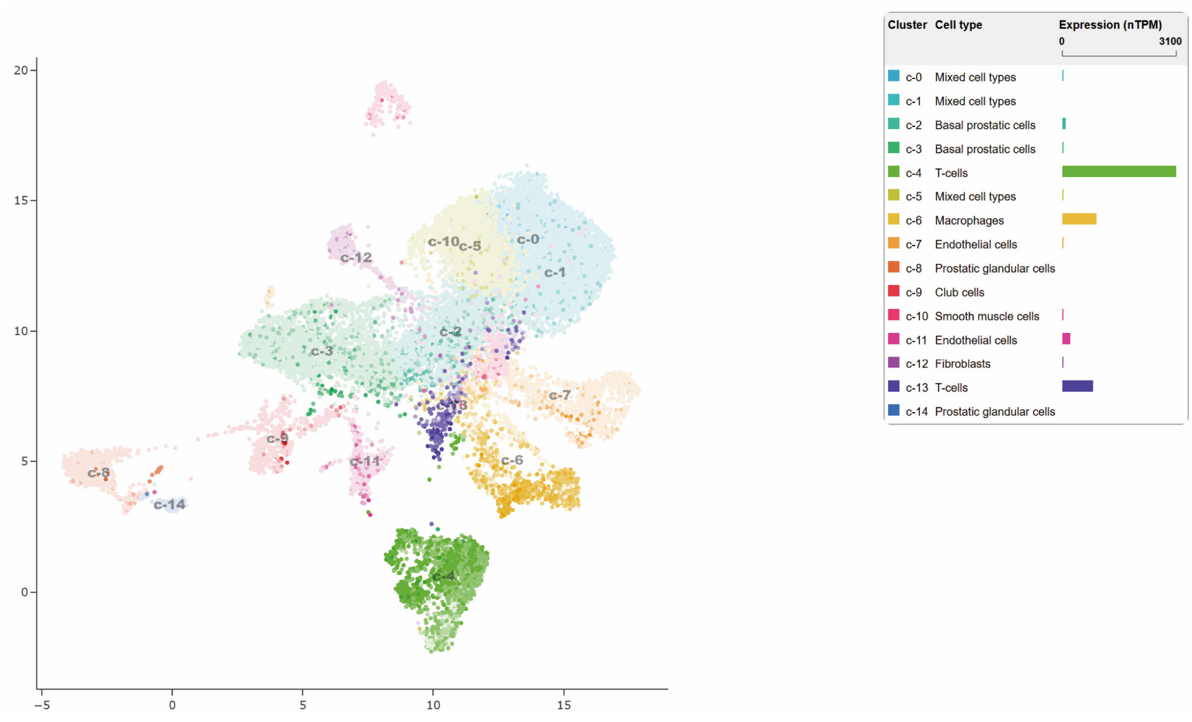
c-0
c-1
c-2
c-3
c-4
c-5
c-6
c-7
c-8
c-9
c-10
c-11
c-12
c-13
c-14
Cluster Cell type Expression (nTPM)
0
3100
c-0 Mixed cell types
c-1 Mixed cell types
c-2 Basal prostatic cells
c-3 Basal prostatic cells
c-4 T-cells
c-5 Mixed cell types
c-6 Macrophages
c-7 Endothelial cells
c-8 Prostatic glandular cells
c-9 Club cells
c-10 Smooth muscle cells
c-11 Endothelial cells
c-12 Fibroblasts
c-13 T-cells
c-14 Prostatic glandular cells

Figure S5

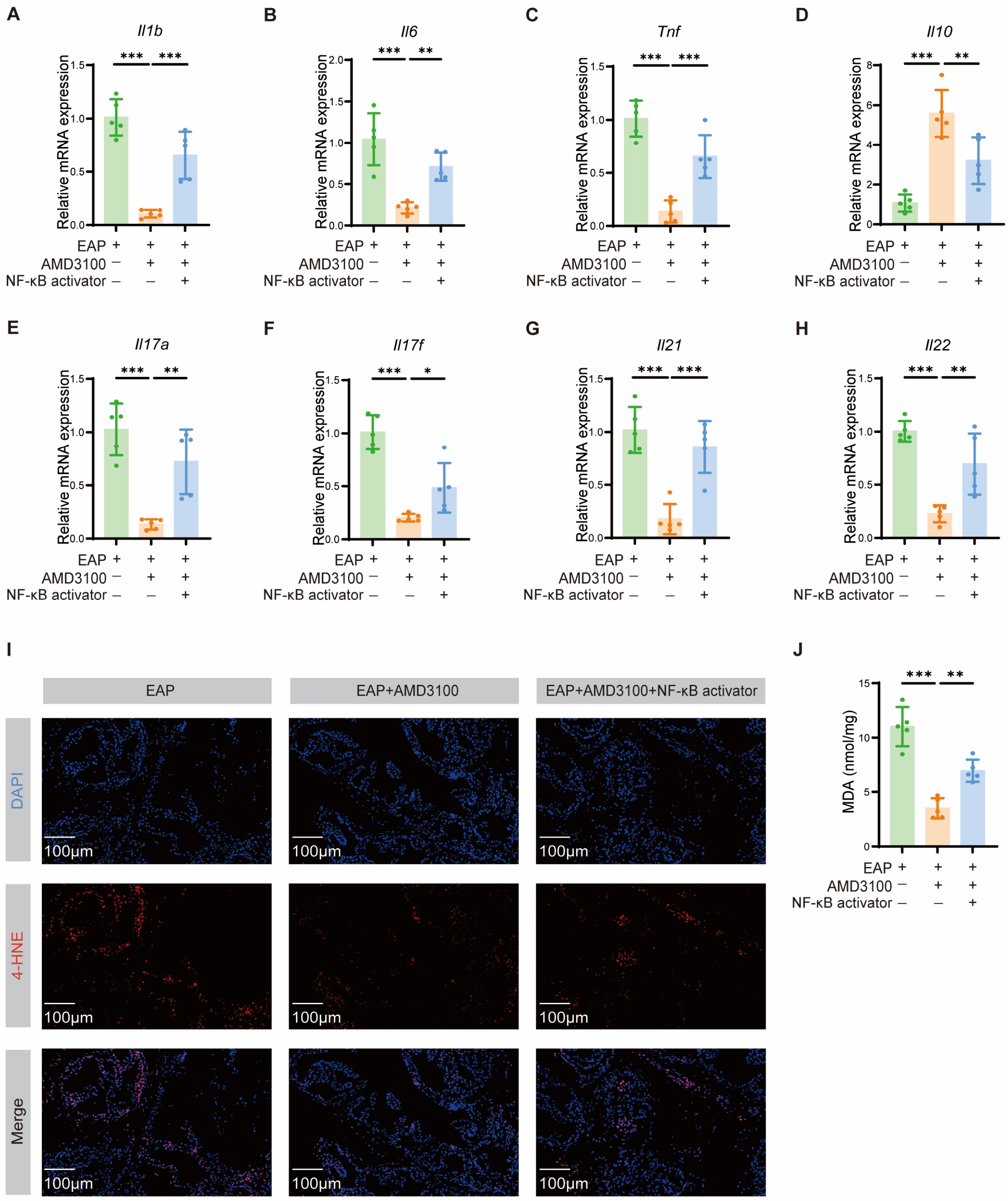

A
Il1b
B
Il6
C
Tnf
D
Il10
E
Il17a
F
Il17f
G
Il21
H
Il22
Relative mRNA expression
EAP
AMD3100
NF-κB activator
I
EAP
EAP+AMD3100
EAP+AMD3100+NF-κB activator
DAPI
4-HNE
Merge
100μm
J
MDA (nmol/mg)
